# Activation of a Copper Biscarbene Mechano‐Catalyst Using Single‐Molecule Force Spectroscopy Supported by Quantum Chemical Calculations

**DOI:** 10.1002/chem.202100555

**Published:** 2021-05-11

**Authors:** Matthew S. Sammon, Michel Biewend, Philipp Michael, Simone Schirra, Milan Ončák, Wolfgang H. Binder, Martin K. Beyer

**Affiliations:** ^1^ Institut für Ionenphysik und Angewandte Physik Universität Innsbruck Technikerstraße 25 6020 Innsbruck Austria; ^2^ Department of Macromolecular Chemistry Martin-Luther-Universität Halle-Wittenberg von-Danckelmann-Platz 4 06120 Halle (Saale) Germany

**Keywords:** computational chemistry, mechanical properties, mechanocatalyst, mechanophore, single-molecule studies

## Abstract

Single‐molecule force spectroscopy allows investigation of the effect of mechanical force on individual bonds. By determining the forces necessary to sufficiently activate bonds to trigger dissociation, it is possible to predict the behavior of mechanophores. The force necessary to activate a copper biscarbene mechano‐catalyst intended for self‐healing materials was measured. By using a safety line bypassing the mechanophore, it was possible to pinpoint the dissociation of the investigated bond and determine rupture forces to range from 1.6 to 2.6 nN at room temperature in dimethyl sulfoxide. The average length‐increase upon rupture of the Cu−C bond, due to the stretching of the safety line, agrees with quantum chemical calculations, but the values exhibit an unusual scattering. This scattering was assigned to the conformational flexibility of the mechanophore, which includes formation of a threaded structure and recoiling of the safety line.

## Graphical abstract

**Point break**: A latent copper‐biscarbene mechanocatalyst becomes active when a ligand is forcefully removed. Pulling with around 2 nN breaks the copper‐carbon bond, as measured by single‐molecule force spectroscopy. To be able to assign the bond rupture event to the Cu−C bond, mechanophores with two different lengths of safety‐line were specifically synthesized for use in this AFM experiment.

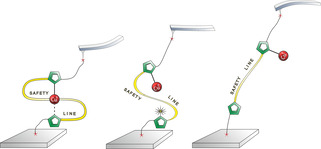

## Introduction

When subjected to mechanical force molecules can experience structural changes, such as bond length and angle distortion, in turn changing the interaction between atoms within the molecule.^[1]^ Depending on how the mechanical force is applied, it is possible to selectively activate certain chemical bonds within a molecule. Hereby, specific reactions can be triggered^[2]^ and utilized in various appli‐cations in stress‐responsive‐ or self‐healing materials as well as mechano‐catalysis,^[3]^ employing specifically designed mechanophores. In many cases the result of mechanochemical processes depends on the mode of activation, often using cavitation[^2^, ^4^] or direct physical stretching of a chemical bond.^[5]^ Ultrasound for example induces indirect stress via cavitation, thus triggering selective chemical reaction pathways,^[6]^ used in metallocene‐activation^[7]^ or subsequent catalysis via indirect activation pathways after Pd‐ or Ag‐ligand cleavage.^[3]^ Over recent years, various mechanophoric metal‐ligand systems have been developed, often triggering catalytic activity of the metal via the now freed coordination site.^[8]^ However, the exact force acting on a mechanophore is difficult to ascertain, as many factors during cavitation or within a material influence the transmittance of the force to the mechanophore, such as the molecules overall rigidity,[^3f^, ^9^] the length of attached residues^[10]^ or the material's morphology.^[11]^ Thus, recent activities on metallocenes,^[7]^ Cu‐pyridine,^[12]^ and B−N bonds^[13]^ demonstrated metal/ligand‐cleavage in polymeric bulk‐systems or in solution, which finally did not allow for a precise, direct quantification of the individual metal/ligand‐bond upon mechanical stressing.

On a microscopic level, the force exerted during a mechanochemically triggered bond dissociation can be measured directly by single‐molecule force spectroscopy (SMFS).^[14]^ In this method a defined force is applied directly to the mechanophore, in turn allowing the accurate determination of the rupture force. The challenge of this approach, however, is to unambiguously assign the observed event to the dissociation of a specific bond within the mechanophore. While some materials show characteristic features upon stretching, e. g. plateaus indicating conformational transitions different from the bond‐rupturing of the mechanophore, such as the chair‐boat‐transition in carboxymethyl amylose (CMA),^[15]^ a chemical design of the mechano‐phore's surrounding is required to address its specific cleavage in SMFS. Craig *et al* synthesized specific polymers of ring‐opening mechanophores, maximizing the amount of activated species in a single experiment. Upon activation, bond dissociation is almost simultaneously triggered in a large number of mechanophores, thus introducing similar characteristics as observed for CMA stretching, most recently employed in the investigation of the palladium carbene bond dissociation.[^4b^, ^16^] A different approach was used by Fernandez *et al*., who exploited chemical activation of stretched disulfide bonds in cystine residues. These act as lock for a protein sequence, keeping it in a folded state. Cleavage of the disulfide bond allows for stretching of the locked protein sequence, leading to a defined length increase.^[17]^

**Figure 1:**
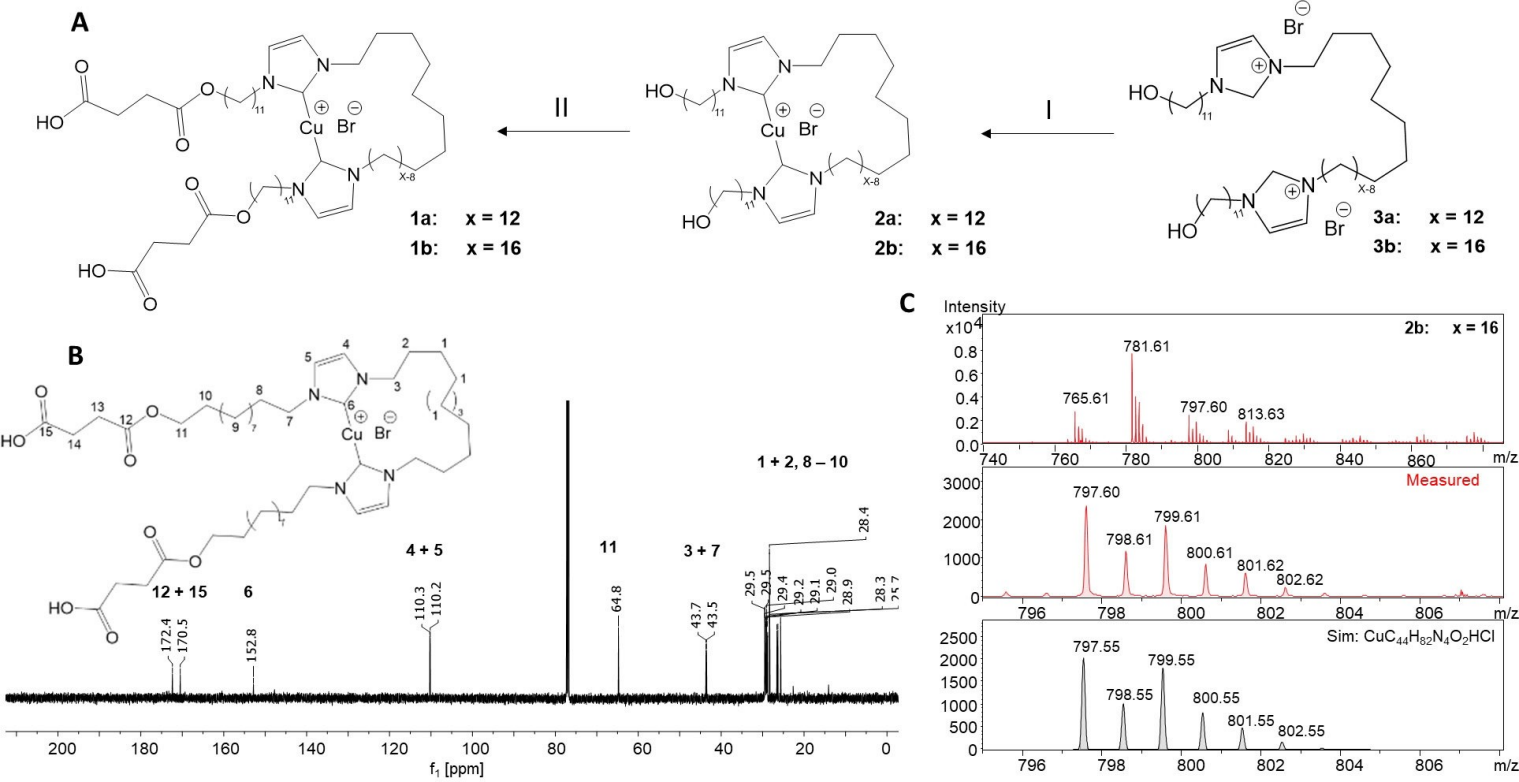
Synthesis for the cyclic bis(NHC)‐Cu(I)‐complex **1 a**, **b**. (A) Synthetic approach starting from **3 a**, **b** via formation of the cyclic bis(NHC)‐Cu(I)‐complex **2 a**, **b**; (I) Cu_2_O/dioxane, 100 °C, 12 hours (**2 a** : 10 %; **2 b** : 16 %); (II) succinic anhydride, triethylamine, THF, 25 °C, (**1 a** : 80 %; **1 b** 72 %). (B) ^13^C‐NMR (CDCl_3_, 100 MHz) expansion of the NHC−C bond‐region of the cyclic complex **1 a**. (C) ESI‐TOF MS of the cyclic intermediate **2 b**.

We here report on SMFS investigation on a bis‐N‐heterocyclic (NHC)‐Cu‐complex, focusing on the direct force required for Cu−C bond rupture in the Cu(I)‐NHC complex. The use of these mechanophoric Cu(I)‐carbene‐complexes,[^3f^, ^9^, ^18^] coupled to a fluorogenic “click”‐reaction, allows to monitor stress‐induced phenomena in linear polymers, semi‐crystalline materials and networks, with an unknown force required to cleave the Cu−NHC− bond. Prior investigation used ultrasound to detach one of the ligands of the copper biscarbene complex [Cu(polymer‐NHC)_2_]X, which creates an open coordination site at the copper center and thus activates the formerly latent catalyst.^[3f]^ Here the complex was tagged with a safety line, connecting the biscarbene ligands and therefore bypassing the copper carbene bonds, given in Figure 1A. The force required for Cu−C bond rupture was measured by dynamic SMFS. The safety line leads to a length increase upon Cu−C bond rupture, which is compared with the results from COnstrained Geometries simulate External Force (COGEF) calculations of the mechanophore. In the case of cyclobutane and triazole mechanophores, this approach allowed us to pinpoint the site of covalent bond rupture.[^14b^, ^19^]

## Results and Discussion

Chemical design of the novel, cyclic bis(NHC)‐Cu(I)‐complexes **1 a**, **b** was accomplished by introducing a safety line between the two NHC‐ligands, complexed to the central Cu(I) metal (Figure 1A). Two different chain length (*x*) were projected, one containing *x*=12 −(CH_2_) segments, one with *x*=16, thus allowing to probe both complexes separately and verify the residual length of the safety line after force‐induced Cu(I)‐NHC‐bond rupture. The synthesis of the two cyclic complexes is described in Figure 1 and the Supporting Information. Starting from the α‐,ω‐alkyl‐bis‐imidazolium bromides (**3 a**, **b**) the direct formation of the cyclic bis‐NHC‐Cu(I)‐complexes **2 a**, **b** was accomplished using Cu_2_O under highly diluted conditions in dioxane to promote formation of the macrocyclic structure. The two complexes were isolated in moderate yields, fully characterized by ^1^H‐NMR, ^13^C‐NMR (see Figure 1B and Supporting Information (S25–S31), and‐in the case of **2 a** and **2 b**‐additionally via ESI‐TOF MS (see Figure 1C, and Figure S36 and S37). It was noted that both complexes were quite labile and it was difficult to obtain proper ESI‐TOF MS spectra, displaying decomposition during ionization. It should be also mentioned that this is one of the first ESI‐characterizations of a cyclic Cu‐bis(NHC)‐complex, from which – unfortunately ‐ single crystals could not be grown. Subsequently, the formation of the complexes **1 a**, **1 b** was proven via ^1^H‐NMR spectroscopy, clearly indicating the attachment of the succinate end groups, useful for subsequent attachment onto the AFM‐tip for the subsequent SFMS‐experiments. Structural analysis of the final complexes **1 a**, **1 b** proved difficult, but not impossible: although a well resolved ^13^C‐NMR spectrum of **1 a** could be easily obtained, this was not possible for the final complex **1 b**. The cyclic complexes **1** and **2** proved sensitive under oxygen, but considerably stable under protective gas, sufficient to enable the AFM experiment. The ESI mass spectra showed no evidence for the formation of dinuclear Cu(bis)‐NHC complexes in significant amounts. The isotopic pattern of the ESI‐TOF spectra assigned to **2 a** and **2 b** do not fit to complexes containing Cu_2_ or Cu_3_ species. No other peak group exhibits an isotopic pattern that would indicate the presence of two Cu atoms in the complex.

**Figure 2:**
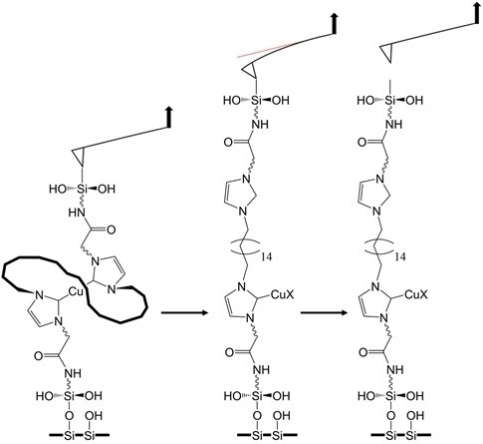
Schematic view of an SMFS measurement, during which bond scission within the mechanophore occurs. Initially (left), the mechanophore is bound between the anchors at surface and cantilever. During the retract (middle), increasing mechanical force leads to scission of the copper carbene bond. Due to the safety line, the connection between cantilever and surface remains intact and the molecule cannot fully relax. By retracting the cantilever further, the mechanical force initiates another bond dissociation, here the detachment of the silane anchor from the cantilever (right).

To determine the forces necessary to activate the catalyst, dynamic SMFS experiments were conducted using atomic force microscopy. Cantilever and surface were functionalized using a silane anchor with a polyethylene glycol spacer and a terminal amine,[^14a^, ^19^] to which the copper biscarbene was bound. Hereby, the desired connection between cantilever and surface was established and upon retraction of the cantilever mechanical force exerted to the molecule. A schematic view is given in Figure 2.

Rupture events from single molecules were identified in the force‐distance curves. From these, possible double ruptures attributed to the breaking of a copper carbene bond and subsequent elongation due to stretching of the safety line could be distinguished. Double rupture events were identified by two characteristic features. The length increase has to correspond to that of the safety line and the slopes of the force‐distance curve before and after the rupture event have to be agree within 4 %. Two types of samples with different lengths of safety lines, a shorter C_12_H_24_ and a longer C16H32 chain, were compared to validate the first criterion. To rule out any bias during data analysis, determination of double rupture events was carried out by two examiners independent of each other and without knowledge of the sample used in the respective measurement‐short safety line, long safety line, no safety line or adipic acid. A comparable number of force curves was obtained for each sample with safety line and references to ensure statistical validity.

**Figure 3:**
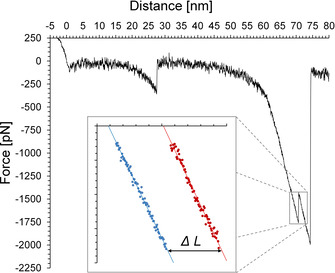
Force‐distance curve of the z‐piezo retract (black) of a single measurement, exhibiting the characteristic features of a double rupture associated with the breaking of the copper carbene bond. The inset shows force plotted against distance before (blue) and after (red) bond dissociation. The length increase ΔL upon rupture is obtained from the difference of the respective linear fits.

**Figure 4:**
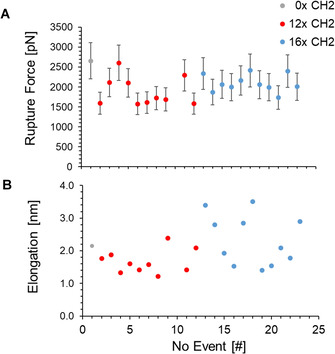
Rupture forces (A) and elongations (B) obtained from curves showing double rupture characteristics. Each dot represents a single measurement. Error bars in A were added in accordance with the widely accepted 20 % margin during these experiments.^[21]^ Data points originating from SMFS experiments with a short (12×CH_2_) safety line are given in red, with a long safety line (16×CH_2_) in blue and the false positive from the reference measurements without a safety line (0×CH_2_) in grey.

Rupture events attributed to the breaking of the copper carbene bond were scarcely identified, showing that the copper carbene bond is significantly stronger and less likely to break under mechanical force than the surface anchors. Since the C−C and C−O bonds in the system are highly stable, the remaining bond breaks are attributed to the siloxane and the amide bonds close to the surface. These bonds are susceptible to force‐induced hydrolysis, as we have shown in previous work,[^14a^, ^14e^, ^20^] and break at forces starting around 800 pN. A typical force‐distance curve with double‐rupture event is given in Figure 3. The forces, at which the double ruptures occur, were highly comparable, ranging from 1.6 to 2.6 nN, Figure 4A. To determine the elongation after rupture of the copper carbene bond, data points before and after the event were plotted and respective linear fits calculated. The elongation was determined by inserting the rupture force into the fit equations and subtracting the distances from another. The obtained elongations for double rupture events are given in Figure 4B. For the shorter safety line, elongations ranging from 1.2 to 2.4 nm and averaging around 1.7 nm were identified. In the case of the longer safety line, the scattering is significantly larger. Elongations from 1.4 to 3.5 nm and on average 2.3 nm were identified, see Table 1.

**Table 1:** Comparison of calculated (calc) and average experimental (exp) rupture forces F and elongations ΔL for short (12×CH_2_) and long (16×CH_2_) safety line as well as difference between average experimental and calculated elongations.

Safety line	Short	Long
*F*_calc_ (0 K)	∼3 nN	
*F*_exp_ (298 K)	1.9 nN	2.1 nN
Δ*L*_calc_	1.6 nm	2.1 nm
Δ*L*_exp_	1.7 nm	2.3 nm
Δ*L*_exp_–Δ*L*_calc_	0.1 nm	0.2 nm

For comparison with experiment, rupture forces were calculated with the COGEF method at the B3LYP/6‐31g* level of theory using Gaussian.^[22]^ Since the strength of the bond is mostly influenced by the close chemical environment of the copper carbene bond, smaller model molecules were used as compromise between computing time and accuracy. Calculated maximum rupture forces at 0 K ranged between 3.0 and 3.3 nN for all models. The presence of solvent and thermal activation, however, significantly lower the actual rupture forces in the experiment. Thus, the measured forces are consistent with the calculations. As shown previously, the scattering over a range of 1 nN is typical for thermally activated bond rupture in force‐ramp experiments.^[23]^ Within the mechanophore, the copper carbene bond was indeed the weakest bond. However, since the silane anchor and the amide bond in the linker are prone to bond dissociation at lower forces,[^14a^, ^14e^, ^20^] it is expected that copper carbene bond dissociation can only rarely be observed, in agreement with experiment.

Recent work by Razgoniaev et al.^[16c]^ revealed rupture forces in the range of 0.8–1.0 nN for the dissociation of the Pd carbene bond during SMFS experiments, which is considerably lower than the forces observed in this work. However, COGEF calculations showed maximum rupture forces of 2.4 nN for the Pd carbene bond as compared to the 3.0–3.3 nN (dependent on the model used) for the Cu carbene bond. Furthermore, retract speeds of 0.3 μm/s were considerably lower than the 1.0 μm/s employed in our experiments, leading to a narrowing of the range at the cost of observable high rupture forces. Therefore, comparison of the measured forces at which dissociation occurs has to focus on the lower end of the rupture force range, showing a difference of 0.8 nN in the experiment, consistent with the difference obtained from COGEF calculations.

**Figure 5:**
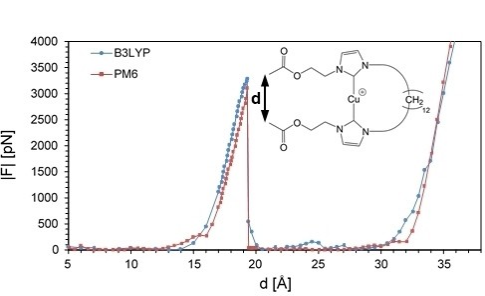
Calculated force‐distance curves of copper biscarbene model molecule with short safety line at B3LYP/6‐31g* (red) and PM6 (blue) level of theory. The force F is given in absolute values, the distance d corresponds to the distance between the terminal C‐atoms. Calculations show an expected elongation of 1.6 nm due to copper carbene bond scission.

For further evaluation, the chemical surroundings within the sample should be taken into consideration, allowing assessment of possible conformational changes. Therefore, model molecules would have to be approximated to the samples used. The significant increase in size of the model molecules in turn is accompanied by a drastic increase of computing time. To balance this size‐dependent increase of computing time, the validity of COGEF calculations using semiempirical methods, viz. PM6, was investigated. Here, rupture forces were slightly lower, ranging between 2.7 and 3.1 nN. The elongations for double ruptures, however, were highly comparable, with differences below 0.1 nm. A typical calculated force distance curve for the short safety line is given in Figure 5. The length increase upon copper carbene bond dissociation was calculated to be between 1.6 and 2.1 nm for the different safety lines, respectively, similar to the average elongations of 1.7 and 2.3 nm obtained from the experiment. An overview is given in Table 1.

Rupture force and average elongation from calculations and the experiment match very well for both lengths of the safety line. There is therefore no doubt that the identified double rupture events result from breaking of the Cu−C bond in the mechanophore. However, the substantial scattering of the measured length increase, in particular for the long safety line, indicates that the situation is more complicated than expected. In our previous studies of the triazol^[19]^ and cyclobutene,^[14b]^ ring‐opening mechanophores, the length increase for the different rupture events showed much smaller variations, deviating at most 0.2 nm from the calculated value. Here, the biggest outlier for the long safety line is 1.2 nm above the average value. The previously studied triazol and cyclobutane mechanophores are rigid, and keep the external pulling points in a fixed position relative to each other and to the safety line. In the present Cu−NHC mechanophore, however, the Cu−C σ‐bonds allow for an almost unhindered rotation of the two NHC units against each other. This internal rotation could allow for partial recoiling of the safety line. The extra large elongations observed with the long safety line require some extra stored length, which is released upon cleavage of a Cu−C bond.

We suggest that a threaded structure can for example be formed when the amine‐terminated PEG anchored at the approaching cantilever slips through the macrocycle before the amide bond is formed, in part directed by the attractive interaction with the copper center. The resulting threaded structure is depicted in Figure S40. A disentanglement of the threaded structure would require to pull the amide and the ester section of the linker through the macrocycle. Even with the long safety line, the size of the macrocycle is at the limit to afford a threaded structure. The more rigid amide or ester units with their extended π electron systems may thus get stuck, and the −(CH_2_)_11_OCO(CH_2_)_2_CONH− section would be available as stored length, to be released upon rupture of a Cu−C bond in addition to the safety line. Such a scenario would explain the outliers in Figure 4B. Since the short safety line does not afford a threaded structure in the first place, this would also explain the absence of these severe outliers in this case.

## Conclusion

Single‐molecule force spectroscopy experiments showed that, while the copper carbene bond is significantly stronger than amide and siloxane anchor, the use of a safety line still allows identification of its mechanochemical dissociation. For the breaking of the copper carbene bond, forces in the range of 1.6–2.6 nN are necessary. The average length increase matches the calculated value for short and long safety line very well, but the individual values exhibit a pronounced scattering for the long safety line. This is attributed to the conformational flexibility of the Cu−NHC mechanophore, in which the Cu−C σ‐bonds allow for almost unhindered rotation of the NHC units, which presumably leads to recoiling and might allow for formation of a threaded structure. With the here presented work we can demonstrate the Cu−NHC‐bond rupture for the first time, allowing to understand previous mechanochemical rupturing of such and similar bonds achieved in bulk materials and solution. It also indicates that the Cu−NHC bond is stronger than expected, opening new room for the molecular and mechanochemical design of such catalytic systems.

## Experimental section

**Synthesis**: The cyclic copper(I) bis(NHC) complexes were prepared via multistep synthesis using bivalent N‐alkyl‐bis‐imidazolium‐ligands, followed by reaction with Cu_2_O. For synthetic details of the Cu(I) bis(NHC) complexes see Supporting Information, Section I) Synthesis and Characterization; for the synthesis of monovalent copper(I) bis(NHC) complexes see ref. [18 a].

**Preparation**: Cantilevers (MLCT, Bruker) were cleaned by irradiation with UV light for 120 min, while glass surfaces were placed in an ultrasonic bath in diluted hydrochloric acid (3.8 %) for 90 min. The glass surfaces were subsequently rinsed and sonicated three more times for 10 min in double distilled water. For functionalization, a mixture of 90 mL Ethanol and 10 mL ultrapure water (HPLC grade) was acidified to pH 4.5–5.5 and 3–5 mg α‐silane‐ω‐amino polyethylene glycol (5 kDa, nanocs) were added and thoroughly mixed. Cleaned glass surfaces and cantilevers were then immersed in this solution for 90 min, rinsed once with water and twice with ethanol and dried in an oven at 60 °C for an hour. After slowly cooling down, glass surfaces and cantilevers were stored under vacuum in a desiccator with silica gel to avoid hydrolysis.

Prior to measurement, cantilever spring constants were determined using the thermal noise method. The terminal carboxylic groups of the copper biscarbene were activated in a solution of non‐dried dimethyl sulfoxide (DMSO) using N′‐(3‐dimethylaminopropyl)‐N‐ethylcarbodiimide hydrochloride (EDC) and N‐hydroxysuccinimide (NHS). Subsequently, the solution with the activated complex was placed on a glass surface, forming amide bonds with the terminal amino group of the polyethylene glycol (PEG) coating.

**SMFS**: To determine the necessary forces to dissociate the copper carbene bond at room temperature, dynamic single‐molecule force spectroscopy (SMFS) experiments were carried‐out using a Nanowizard 4 by JPK. Reference measurements were performed using adipic acid or a copper‐biscarbene complex without safety line in order to accurately identify double rupture events. A detailed description of the procedure is given in the Supporting Information, Section II) Single Molecule Force Spectroscopy.

Dynamic SMFS experiments were conducted in DMSO and by approaching the PEG coated cantilever tip to the surface (Setpoint 250 pN) and keeping it in close contact for 3 s, allowing another amide bond to form between coated cantilever and activated biscarbene. By retracting the cantilever from the surface at constant velocity (1 μm/s), increasing mechanical force was applied to the system, leading to bond ruptures and cantilever detachment. The cantilever was then moved to the next point on a predefined measurement grid and the experiment was repeated.

Around 15–20 % of the approaches resulted in covalent attachment of the mechanophore and about 1 % of these force‐distance curves exhibited characteristic double rupture features. From reference measurements, a single force‐distance curve out of 16,000 showed the characteristics of a double‐rupture, being a false positive result. By comparison, 21 double rupture events were observed from 44,400 measurements using copper complexes with safety lines, i. e. 1 in 2114 force curves resulted in a double rupture event with safety line, an 8‐fold increase compared to 1 in 16,000 without safety line. No double rupture events were seen using adipic acid instead of Cu‐biscarbene complexes, clearly showing that characteristic bond dissociation originates from biscarbene complexes.

**Quantum chemical calculations**: Using quantum chemical calculations, the behavior of the copper biscarbene complexes upon exposure to mechanical force was simulated. COGEF calculations^[24]^ were carried out at 0.5 Å step size for lower forces and 0.1 Å step size close to rupture events. The expected maximum rupture forces were calculated for smaller model molecules at the B3LYP/6‐31(+)g* level of theory. To determine the elongation upon rupture of the copper carbene bond, simulations were carried‐out for the actual complex used in the experiments. Due to the size of the complex, the semi‐empirical PM6 approach was chosen. All calculations were performed in the Gaussian software.

## Conflict of interest

The authors declare no conflict of interest.

## Supporting information

As a service to our authors and readers, this journal provides supporting information supplied by the authors. Such materials are peer reviewed and may be re‐organized for online delivery, but are not copy‐edited or typeset. Technical support issues arising from supporting information (other than missing files) should be addressed to the authors.

Supplementary
