# Supplementary material for: Activation of a Copper Biscarbene Mechano‐Catalyst Using Single‐Molecule Force Spectroscopy Supported by Quantum Chemical Calculations
Source: Chemistry. 2021 May 11;27(34):8723–9. doi: 10.1002/chem.202100555 (PMC8251802; doi:10.1002/chem.202100555)

Chemistry–A European Journal

Supporting Information

Activation of a Copper Biscarbene Mechano-Catalyst Using Single-Molecule Force Spectroscopy Supported by Quantum Chemical Calculations

Matthew S. Sammon, Michel Biewend, Philipp Michael, Simone Schirra, Milan Ončák, Wolfgang H. Binder,* and Martin K. Beyer*

Content

I) Synthesis and Characterization
- General Information
- Synthesis
- NMR and ESI Spectra

II) Single-Molecule Force Spectroscopy
- Consumables / Chemicals
- Functionalization Procedure
- Experiment
- Data Analysis

III) Computation

I) Synthesis and Characterization

General Information

All reactions were carried out under dry, inert argon atmosphere using common Schlenk techniques unless noted otherwise. All solvents were purchased in technical grade and were distilled before further use, while dry solvents were prepared using standard drying techniques and were subsequently degassed by freeze-pump-thaw-cycles. The chemicals were purchased from Sigma-Aldrich or VWR and used as received. *N*-methylimidazole was dried over molecular sieve and distilled after purchase. NMR spectra were recorded on a Varian Gemini 400 or 500 spectrometer at 27°C. Chemical shifts (δ) are reported in ppm and referred to the solvent residual signal ($CDCl_3$ 7.26 ppm for ^1H and 77.0 ppm for ^{13}C; THF-d_8 3.58 and 1.72 ppm for ^1H as well as 67.2 and 25.3 ppm for ^{13}C; DMSO-d_6 2.50 ppm for ^1H and 39.5 ppm for ^{13}C).

ESI-TOF-MS measurements were performed on a Bruker Daltonics microTOF via direct injection with a flow rate of 180 µL h^{-1} using the positive or negative mode with an acceleration voltage of 4.5 kV. Samples were prepared by dissolving sample in HPLC grade solvent at a concentration of 1 mg mL^{-1} without additional salt.

Synthesis

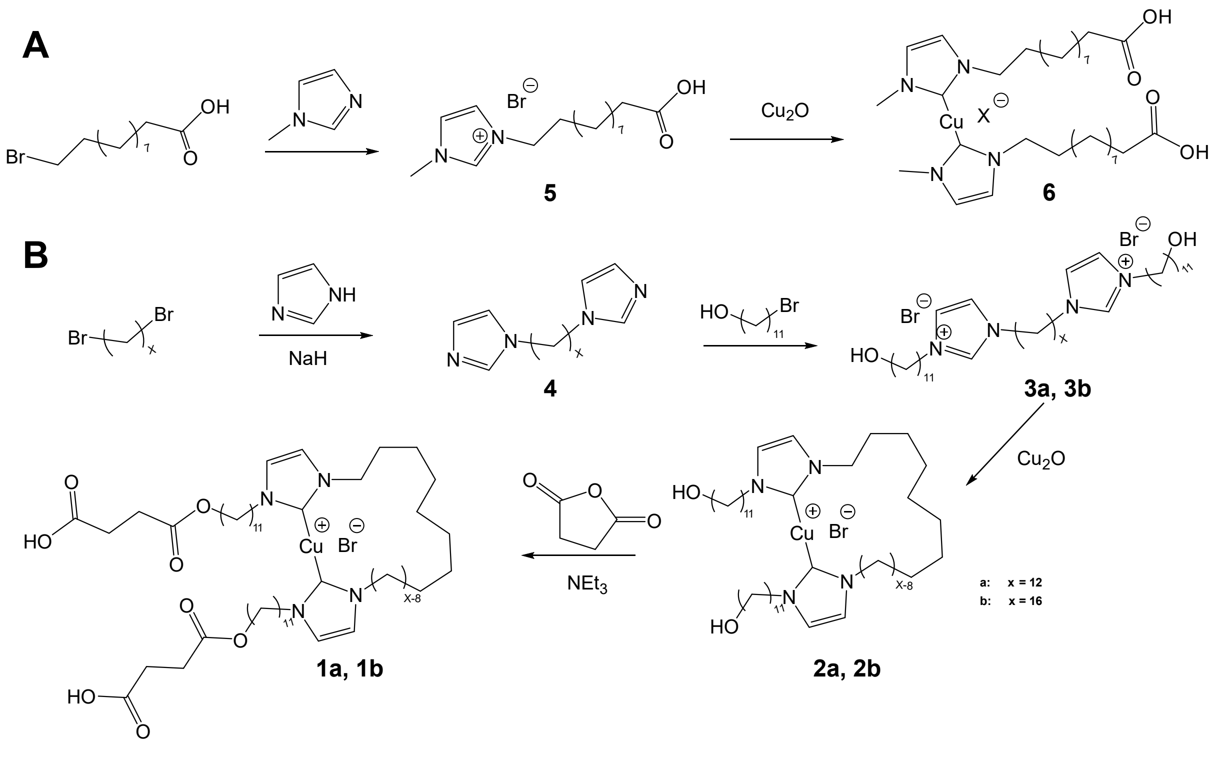

*Figure S1: A: Two step synthesis of acyclic copper(I) bis(NHC) complex by direct quaternization with subsequent complexation reaction towards COOH-functionalized (**6**) Cu-bis(NHC) complexes applying copper(I)-oxide*
*B: Synthetic route for cyclic complexes with different chain length (**1a** for x=12 and **1b** for x=16).*

1.1.1. Synthesis of 3-(10-carboxydecyl)-1-methylimidazolium bromide (**5**)

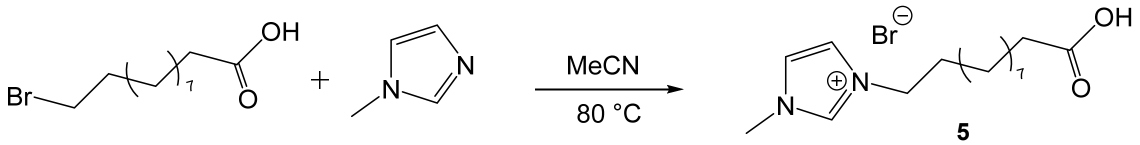

*Figure S2: Synthesis of 3-(10-carboxydecyl)-1-methylimidazolium bromide (**5**).*

1-Methylimidazole (1.50 mL, 19 mmol) was added to a suspension of 11-bromoundecanoic acid (5.30 g, 20 mmol) in acetonitrile (MeCN) (20 mL) at room temperature (RT) and was stirred for 24 h at 80°C. The reaction mixture was allowed to come to RT and was diluted with diethyl ether (Et_2O) (80 mL). The resulting precipitate was filtered off, washed with Et_2O (3 x 50 mL) and **5** was obtained as colorless solid after drying in high vacuum (6.31 g, 18.17 mmol, 95%).

^{1}H-NMR (DMSO-d_6, 400 MHz): δ [ppm] 11.94 (*s*, 2H, COO*H*), 9.13 (*s*, 2H, NC*H*N), 7.76 (*s*, 2H, NC*H*CHN), 7.69 (*s*, 2H, NCHC*H*N), 4.14 (*t*, $^3J_{H,H}$ = 7.2 Hz, 4H, NCH_2), 3.83 (*s*, 6H, NCH_3), 2.16 (*t*, $^3J_{H,H}$ = 7.3 Hz, 4H, OCH_2), 1.75 (*m*, 4H, NCH$_2$CH_2), 1.46 (*m*, 4H, OCH$_2$CH_2), 1.22 (*m*, 24H, (CH_2)$_6$).

^{13}C NMR (DMSO-d_6, 100 MHz): δ [ppm] 174.9 (*C*OOH), 137.0 (NCHN), 124.0 (CH$_3$N*C*HCHN), 122.7 (CH$_3$NCH*C*HN), 49.2 (N*C*H$_2$), , 36.2 (N*C*H$_3$), 34.1 (O*C*H$_2$), 29.8 (OCH$_2$*C*H$_2$), 29.2, 29.2, 29.1, 29.0, 28.8, 25.9, 24.9.

ESI-TOF MS (positive mode, MeOH, *m/z*) [M-Br]$^+$ found 267.206, simulated 267.207 for $C_{15}H_{27}N_2O_2^+$.

1.1.2. Synthesis of COOH end-capped Cu(I) bis(NHC) complex (**6**)

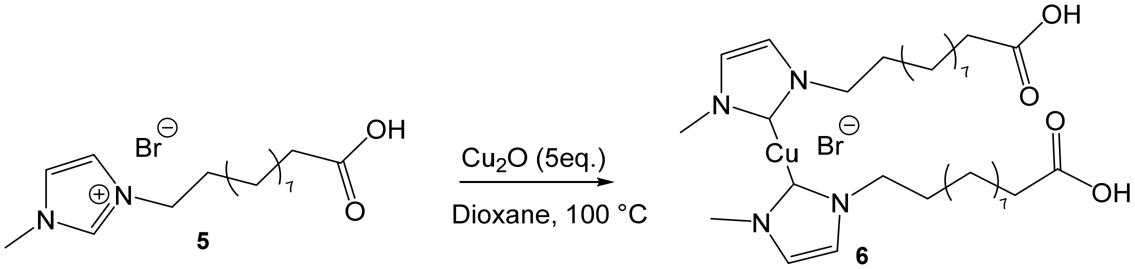

*Figure S3: Synthesis of COOH end-capped Cu(I) bis(NHC) complex (**6**).*

Compound **5** (2.08 g, 6.0 mmol) and copper(I)-oxide (4.30 g, 30.0 mmol, 5 eq.) were suspended in dioxane (50 mL) and stirred at 100°C for 3 days. After cooling to RT, the excess of Cu_2O was filtered off and the solvent was removed under reduced pressure. Compound **6** (0.54 g, 0.8 mmol, 13%) was obtained as a white solid after column chromatography on silica by gradually changing the polarity of solvent from pure $CHCl_3$ to $CHCl_3$/MeOH 10/1 (R_f = 0.24 $CHCl_3$/MeOH 20/1).

^{1}H-NMR (CDCl$_3$, 400 MHz): δ [ppm] 6.18 (q, 4H, NC*H*C*H*N), 3.60 (t, $^3J_{H,H}$ = 7.3 Hz, 4H, NCH_2), 3.12 (s, 6H, NCH$_3$), 2.34 (t, $^3J_{H,H}$ = 7.2 Hz, 4H, OCCH_2), 1.71 – 1.58 (m, 8H, NCH$_2$CH_2), 1.31 (m, 24H, (CH_2)$_{12}$).

13C-NMR (CDCl3, 100 MHz): δ [ppm] 174.3 (*C*=O), 153.2 (N*C*CuN,) 111.0 (CH_3NCHCHN), 109.9 (CH_3N*C*HCHN), 51.4 (N*C*H_2), 43.6 (N*C*H_3), 34.1 (O*C*H_2), 30.3, 29.5, 29.4, 29.3, 29.5, 29.1, 28.1, 26.6, 24.9.

1.1.3. Synthesis of 1,16-dibromohexadecane

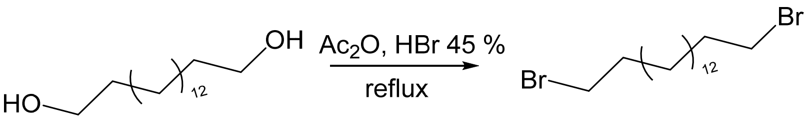

Figure S4: Synthesis of 1,16-dibromohexadecane.

HBr (48% in H_2O, 63.0 mL, 380 mmol) was added dropwise to Ac_2O (108 mL, 1.1 mol) at 0°C, followed by the addition of compound 1,16-dihydroxyhexadecan (4.47 g, 17 mmol). The reaction mixture was stirred at reflux 24 h. After cooling to RT, the solution was extracted with hexane (3 x 50 mL) and washed with H_2O (3 x 100 mL). The solvent was removed under reduced pressure and 1,16-dibromohexadecane (17 mmol, 6.55 g, 99%) was obtained as a white solid.

<u>1H-NMR (CDCl3, 400 MHz):</u> δ [ppm] 3.41 (t, $^3J_{H,H}$ = 6.9 Hz, 4H, BrC*H_2*), 1.85 (p, $^3J_{H,H}$ = 6.9 Hz, 4H, BrCH2C*H_2*), 1.48 – 1.36 (m, 4H, BrCH2CH2C*H_2*), 1.27 (m, 20H, (C*H_2*)10).

<u>13C-NMR (CDCl3, 100 MHz):</u> δ [ppm] 34.0 (Br*C*H2), 32.8 (BrCH2*C*H2), 29.6 (Br(CH2)2*C*H2), 29.6 (Br(CH2)3*C*H2), 29.5 (Br(CH2)4*C*H2), 29.4 (Br(CH2)5*C*H2), 28.8 (Br(CH2)6*C*H2), 28.2 (Br(CH2)7*C*H2).

1.1.4. Synthesis of ω,n-di(imidazolyl) alkane (**4**)

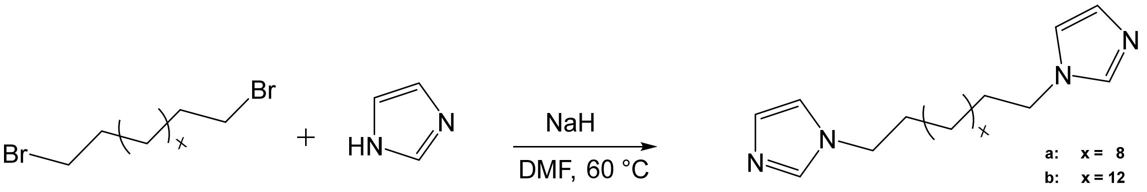

4

*Figure S5: Synthesis of ω,n-di(imidazolyl) alkane (**4**) with different chain length.*

NaH (80.0 mmol, 60%) was suspended in DMF (50 mL) and was cooled to 0°C followed by the dropwise addition of imidazole (72.4 mmol). After stirring for 15 min at 0°C α,ω-dibromoalkane (30.0 mmol) was added, the reaction mixture was then heated up to 70°C and stirred for additional 4 h. The reaction mixture was allowed to reach RT, was diluted with ethylacetate (120 mL) and was washed with water (3 x 50 mL). The combined organic phases were dried via $NaSO_4$ and after the solvent was removed under reduced pressure compound **4** was obtained as colorless solid.

<u>Characterization of **4a** (X = 8):</u>

<u>Yield:</u> (8.60 g, 28.5 mmol, 95%)

<u>1H-NMR (CDCl3, 400 MHz):</u> δ [ppm] 7.46 (s, 2H, NC*H*N), 7.06 (s, 2H, CH2NC*H*CHN), 6.90 (s, 2H, CH3NC*H*CHN), 3.91 (t, $^3J_{H,H}$ = 7.1 Hz, 4H, NC*H_2*), 1.76 (p, $^3J_{H,H}$ = 6.8 Hz, 4H, NCH2C*H_2*), 1.36 – 1.18 (m, 16H, (C*H_2*)8).

13C-NMR (CDCl3, 100 MHz): δ [ppm] 137.0 (NCHN), 129.4 (NCHCHN), 118.7 (NCHCHN), 47.0 (NCH_2), 31.0 (NCH_2CH_2), 29.4 ($N(CH_2)_2CH_2$), 29.3 ($N(CH_2)_3CH_2$), 29.0 ($N(CH_2)_4CH_2$), 26.5 ($N(CH_2)_5CH_2$).

ESI-TOF MS (positive mode, THF, *m/z*): $[M+Na]^+$ found 325.235, simulated 325.236 for $C_{18}H_{30}N_4Na^+$.

Characterization of **4b** (X = 12):

Yield: (9.98 g, 27.9 mmol, 93%)

^{1}H-NMR (DMSO-d_6, 400 MHz): δ [ppm] 7.58 (s, 2H, NC*H*N), 7.13 (s, 2H, CH_2NC*H*CHN), 6.85 (s, 2H, CH_3NC*H*CHN), 3.91 (t, $^3J_{H,H}$ = 7.1 Hz, 4H, NCH_2), 1.66 (p, $^3J_{H,H}$ = 7.2 Hz, 4H, NCH_2CH_2), 1.20 (m, 24H, $(CH_2)_{12}$).

^{13}C-NMR (DMSO-d_6, 125 MHz): δ [ppm] 137.7 (NCHN), 128.7 (NCHCHN), 119.7 (NCHCHN), 46.4 (NCH_2), 40.0 (NCH_2CH_2), 31.0 ($N(CH_2)_2CH_2$), 29.5 ($N(CH_2)_3CH_2$), 29.4 ($N(CH_2)_4CH_2$), 29.6 ($N(CH_2)_5CH_2$), 28.9 ($N(CH_2)_6CH_2$), 26.3 ($N(CH_2)_7CH_2$).

ESI-TOF MS (positive mode, THF, *m/z*): $[M+H]^+$ found 359.316, simulated 359.317 for $C_{22}H_{39}N_4O_2^+$.

1.1.5. Synthesis of 1,1'-(alkane-1, ω-diyl)bis(3-(hydroxyundecyl)imidazolium) bromide (**3**)

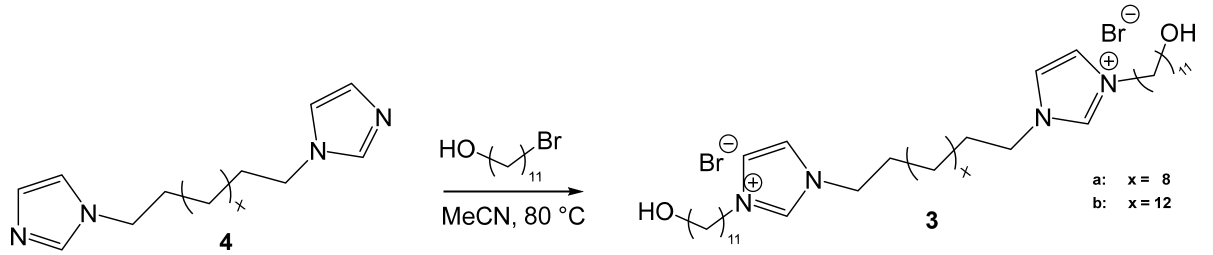

*Figure S6: Synthesis of 1,1'-(alkane-1,ω-diyl)bis(3-(11-hydroxyundecyl)imidazolium) bromide (**3**) with different chain length.*

Compound **4** (13.25 mmol) was added to a suspension of 11-bromoundecan-1-ol (7.50 g, 30.00 mmol) in acetonitrile (MeCN) (100 mL) at RT and was stirred for 24 h at 80°C. The reaction mixture was allowed to come to RT and was diluted with diethyl ether (Et_2O) (120 mL). The resulting precipitate was filtered off, washed again with Et_2O (3 x 50 mL) and **3** was obtained as colorless solid after drying in high vacuum.

Characterization of **3a** (X = 8):

Yield: (10.46 g, 13.0 mmol, 98%)

^{1}H-NMR (DMSO-d_6, 500 MHz): δ [ppm] 9.21 (s, 2H, NC*H*N), 7.80 (s, 4H, NC*H*C*H*N), 4.30 (t, $^3J_{H,H}$ = 5.1 Hz, 2H, O*H*), 4.15 (t, $^3J_{H,H}$ = 7.0 Hz, 8H, $CH_2NCHNCH_2$), 3.36 (q, $^3J_{H,H}$ = 6.4 Hz, 4H, OCH_2), 1.77 (m, 8H, $CH_2CH_2NCHNCH_2CH_2$), 1.38 (p, $^3J_{H,H}$ = 6.7 Hz, 4H, OCH_2CH_2), 1.29 – 1.11 (m, 44H, $(CH_2)_{22}$).

^{13}C-NMR (DMSO-d_6, 125 MHz): δ [ppm] 136.4 (NCN), 122.9 (NCHCHN), 61.2 (OCH_2), 49.3 (NCH_2), 40.6, 40.4, 40.2, 40.0, 39.9, 33.0, 29.7, 29.7, 29.5, 29.5, 29.4, 29.4, 29.3, 29.3, 28.9, 28.8, 26.0, 26.0, 25.9.

ESI-TOF MS (positive mode, THF, *m/z*): $[M-2Br]^{2+}$ found 322.299, simulated 322.298 for $C_{40}H_{76}N_4O_2^{2+}$.

Characterization of **3b** (X = 12):

Yield: (10.85 g, 12.6 mmol, 95%)

^{1}H-NMR (DMSO-d_6, 400 MHz): δ [ppm] 9.19 (s, 2H, NC*H*N), 7.78 (s, 4H, NC*H*C*H*N), 4.29 (t, $^3J_{H,H}$ = 5.1 Hz, 2H, O*H*), 4.14 (t, $^3J_{H,H}$ = 7.0 Hz, 8H, CH_2NCHNCH_2), 3.36 (q, $^3J_{H,H}$ = 6.4 Hz, 4H, OCH_2), 1.78 (p, $^3J_{H,H}$ = 7.4 Hz, 8H, CH_2CH$_2$NCHNCH$_2$CH_2) 1.37 (p, $^3J_{H,H}$ = 6.8 Hz, 4H, OCH$_2$CH_2), 1.28 – 1.13 (m, 52H, (CH_2)$_{26}$).

^{13}C-NMR (DMSO-d_6, 125 MHz): δ [ppm] 136.4 (N*C*N), 122.9 (N*C*H*C*HN), 61.1 (O*C*H$_2$), 49.3 (N*C*H$_2$), 40.4, 40.2, 40.0, 39.8, 39.6, 33.0, 29.7, 29.6, 29.6, 29.5, 29.5, 29.4, 29.3, 29.3, 28.8, 28.8, 26.0, 25.90.

ESI-TOF MS (positive mode, THF, *m/z*): [M-2Br]$^{2+}$ found 350.327, simulated 350.329 for $C_{44}H_{84}N_4O_2^{2+}$.

1.1.6. Synthesis of OH end-capped Cu(I) bis(NHC) complexes with a safety line (**2a, 2b**)

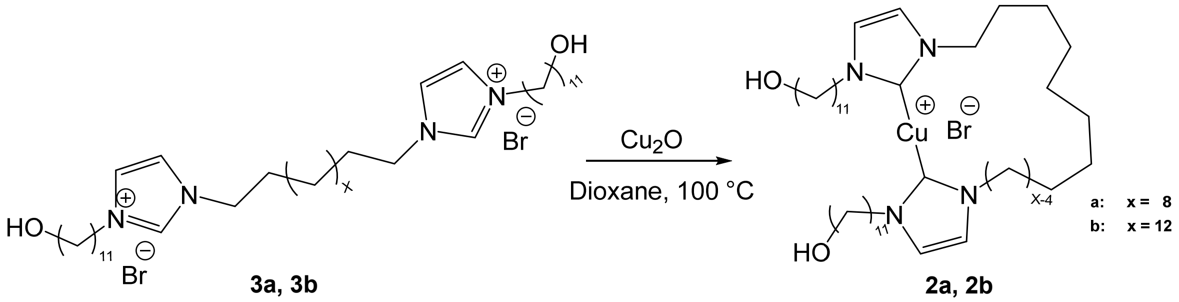

*Figure S7: Synthesis of OH end-capped Cu(I) bis(NHC) complexes (**2a, 2b**) with different length of safety line using the copper(I) oxide method.*

Compound **3** (6.00 mmol) and copper(I)-oxide (4.3 g, 30.00 mmol, 5 eq.) were suspended in dioxane (300 mL) and the reaction mixture was stirred at 100°C overnight. After cooling to RT, the excess of Cu_2O was filtered off and the solvent was removed under reduced pressure. Compound **2** was obtained after column chromatography on silica by gradually changing the polarity of solvent from pure $CHCl_3$ to $CHCl_3$/MeOH 20/1 (R_f = 0.42 $CHCl_3$/MeOH 20/1) as a light-yellow solid.

Characterization of **2a** (X = 8):

Yield: (0.40 g, 0.60 mmol, 10%)

^{1}H-NMR (THF-d_8, 400 MHz): δ [ppm] 6.26 (s, 4H, NC*H*C*H*N), 3.52 (t, $^3J_{H,H}$ = 7.1 Hz, 8H, CH_2NCHNCH_2), 3.46 (q, $^3J_{H,H}$ = 5.9 Hz, 4H, OCH_2), 3.42 (t, $^3J_{H,H}$ = 4.8 Hz, 2H, O*H*), 1.65 – 1.56 (m, 8H, CH_2CH$_2$NCHNCH$_2$CH_2), 1.47 (p, $^3J_{H,H}$ = 6.8, 4H, OCH$_2$CH_2), 1.41 – 1.22 (m, 44H, (CH_2)$_{22}$).

^{13}C-NMR (THF-d_8, 100 MHz): δ [ppm] 152.9 (N*C*CuN), 109.9 (N*C*HCHN), 109.9 (NCH*C*HN), 63.0 (O*C*H$_2$), 43.5 (N*C*H$_2$), 43.4 (N*C*H$_2$), 32.8, 29.7, 29.5, 29.5, 29.5, 29.4, 29.3, 29.2, 29.1, 26.6, 26.5, 25.7.

ESI-TOF MS (positive mode, THF, *m/z*): [M + CH_3OH]$^+$ found 737.138, simulated 737.538 for $C_{41}H_{77}CuN_4O_3^+$

Characterization of **2b** (X = 12):

Yield: (0.73 g, 0.96 mmol, 16%)

^{1}H-NMR (THF-d_8, 400 MHz): δ [ppm] 6.24 (s, 4H, NC*H*C*H*N), 3.51 (t, $^3J_{H,H}$ = 7.1 Hz, 8H, C*H₂*NCHNC*H₂*), 3.46 (q, $^3J_{H,H}$ = 6.4 Hz, 4H, OC*H₂*), 3.34 (t, $^3J_{H,H}$ = 5.2 Hz, 2H, O*H*), 1.60 (p, $^3J_{H,H}$ = 7.3 Hz, 8H, C*H₂*CH₂NCHNCH₂C*H₂*), 1.46 (p, $^3J_{H,H}$ = 6.7 Hz, 4H, OCH₂C*H₂*), 1.38 – 1.13 (m, 52H, (C*H₂*)₂₆).

^{13}C-NMR ($CDCl_3$, 100 MHz): δ [ppm] 152.9 (N*C*CuN), 109.9 (N*C*HCHN), 109.8 (NCH*C*HN), 63.0 (O*C*H₂), 43.5 (N*C*H₂), 43.4 (N*C*H₂), 32.8, 29.6, 29.6, 29.5, 29.5, 29.5, 29.5, 29.4, 29.3, 29.2, 29.1, 26.6, 26.6, 25.7.

ESI-TOF MS (positive mode, THF, *m/z*): $[M-Br+Cl+H]^+$ found 797.605, simulated 797.549 for $C_{44}H_{83}CuN_4O_2Cl^+$

1.1.7. Synthesis of COOH end-capped Cu(I) bis(NHC) complexes with safety line (**1a, b**)

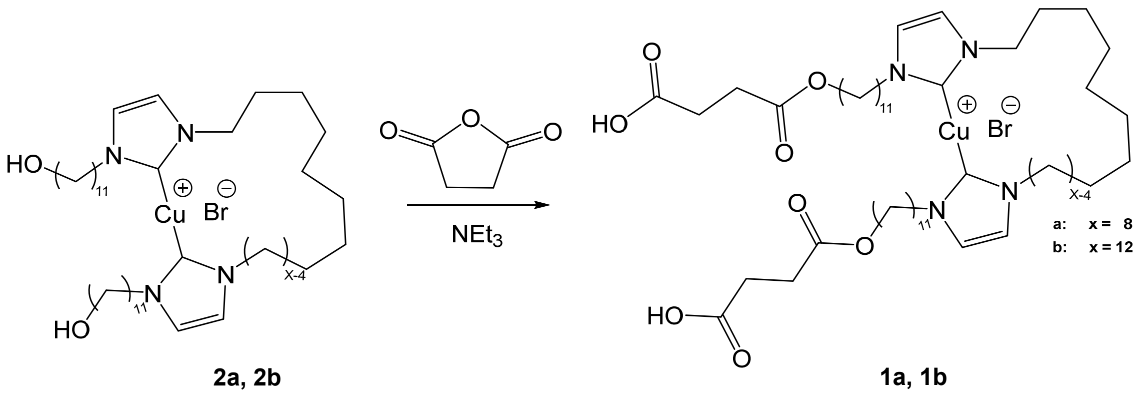

*Figure S8: Synthesis of COOH end-capped Cu(I) bis(NHC) complexes (**1a, 1b**) with different length of safety line.*

Compound **2** (0.15 mmol) was dissolved in THF (5 mL) followed by the addition of NEt_3 (50 μL, 0.35 mmol). The reaction mixture was stirred at RT overnight, washed with HCl_{aq} (10 mL, 1M) and was extracted with CH_2Cl_2 (3 x 20 mL). The combined organic phases were concentrated under reduced pressure and the compound **1** was obtained after precipitation with hexane as a colorless solid (R_f = 0.37 $CHCl_3$/MeOH 20/1).

Characterization of **1a** (X = 8):

Yield: (0.18 g, 0.12 mmol, 80%)

^{1}H-NMR ($CDCl_3$, 500 MHz): δ [ppm] 6.19 (s, 4H, NC*H*C*H*N), 4.10 (t, $^3J_{H,H}$ = 6.3 Hz, 4H, OC*H₂*), 3.61 (m, 8H, C*H₂*NCHNC*H₂*), 2.64 (m, 8H, OCC*H₂*C*H₂*CO), 1.63 (m, 12H, C*H₂*CH₂NCHNCH₂C*H₂* + OCH₂C*H₂*), 1.40 – 1.07 (m, 36H, (C*H₂*)₁₈).

^{13}C-NMR ($CDCl_3$, 100 MHz): δ [ppm] 172.4 (HO*C*=O), 170.5 (CH₂O*C*=O), 152.8 (N*C*CuN), 110.3 (CH₃NCH*C*HN), 110.2 (CH₃N*C*HCHN), 64.8 (O*C*H₂), 43.7 (N*C*H₂), 43.5 (N*C*H₂), 29.5 (HOC=O*C*H₂), 29.5 (CH₂OC=O*C*H₂), 29.4, 29.2, 29.1, 29.0, 29.0, 28.9, 28.8, 28.4, 28.3, 26.5, 26.3, 25.7.

Characterization of **1b** (X = 12):

Yield: (0.11 g, 0.10 mmol, 72%)

<u>^{1}H-NMR (CDCl$_3$, 500 MHz):</u> δ [ppm] 6.19 (s, 4H, NC*H*C*H*N), 4.10 (t, $^3J_{H,H}$ = 6.3 Hz, 4H, OC*H$_2$*), 3.61 (td, J = 7.3, 2.9 Hz, 8H, C*H$_2$*NCHNC*H$_2$*), 2.64 (dq, J = 10.6, 5.8 Hz, 8H, OCC*H$_2$*C*H$_2$*CO), 1.63 (m, 12 H, C*H$_2$*CH$_2$NCHNCH$_2$C*H$_2$* + OCH$_2$C*H$_2$*), 1.39 – 1.20 (m, 44H, (C*H$_2$*)$_{22}$).

<u>^{13}C-NMR (CDCl$_3$, 100 MHz):</u> δ [ppm] 172.4 (HO*C*=O), 170.5 (CH$_2$O*C*=O), 152.8 (N*C*CuN), 110.3 (CH$_3$NCH*C*HN), 110.2 (CH$_3$N*C*HCHN), 64.8 (O*C*H$_2$), 43.7 (N*C*H$_2$), 43.5 (N*C*H$_2$), 29.5 (HOC=O*C*H$_2$), 29.5 (CH$_2$OC=O*C*H$_2$), 29.4, 29.2, 29.1, 29.0, 29.0, 28.9, 28.9, 28.8, 28.8, 28.6, 28.5, 28.4, 28.3, 26.5, 26.3, 25.7.

1.1.8. Synthesis of 3-(11-hydroxyundecyl)-1-hexylimidazolium bromide

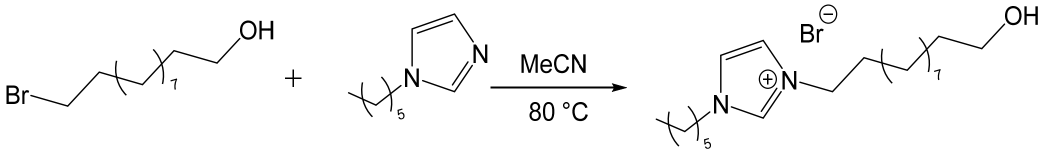

Figure S9: Synthesis of 3-(11-hydroxyundecyl)-1-hexyllimidazolium bromide.

1-Hexylimidazole (1.52 mL, 10.00 mmol) was added to a suspension of 11-bromoundecan-1-ol (2.38 g, 9.50 mmol) in acetonitrile (MeCN) (20 mL) at room temperature (RT) and was stirred for 24 h at 80°C. The reaction mixture was allowed to come to RT and was diluted with diethyl ether (Et$_2$O) (120 mL). The resulting precipitate was filtered off, washed with Et$_2$O (3 x 50 mL) and 3-(11-hydroxyundecyl)-1-hexyllimidazolium bromide was obtained as colorless solid after drying in high vacuum (3.75 g, 9.30 mmol, 98%).

<u>^{1}H-NMR (DMSO-*d*$_6$, 500 MHz):</u> δ [ppm] 9.21 (*s*, 1H, NC*H*N), 7.79 (*m*, 2H, NC*H*C*H*N), 4.29 (*t*, $^3J_{H,H}$ = 5.1 Hz, 1H, O*H*), 4.14 (*t*, $^3J_{H,H}$ = 7.0 Hz, 4H, NC*H$_2$*), 3.36 (*q*, $^3J_{H,H}$ = 5.4 Hz, 2H, OC*H$_2$*), 1.77 (*p*, $^3J_{H,H}$ = 7.2 Hz, 4H, NCH$_2$C*H$_2$*), 1.37 (*p*, $^3J_{H,H}$ = 6.7 Hz, 2H, OCH$_2$C*H$_2$*), 1.28 – 1.15 (*m*, 20H, (C*H$_2$*)$_{10}$), 0.84 (*t*, $^3J_{H,H}$ = 6.9 Hz, 3H, C*H$_3$*).

<u>^{13}C NMR (DMSO-*d*$_6$, 125 MHz):</u> δ [ppm] 136.4 (NCHN), 122.9 (N*C*H*C*HN), 61.1 (O*C*H$_2$), 49.3 (N*C*H$_2$), 32.9 (OCH$_2$*C*H$_2$), 30.9, 29.7, 29.7, 29.5, 29.4, 28.8, 26.0, 25.9, 25.6, 22.3, 14.24 (*C*H$_3$).

<u>ESI-TOF MS (positive mode, MeOH, *m/z*):</u> [M-Br]$^+$ found 323.304, simulated 323.306 for $C_{20}H_{39}N_2O^+$.

1.1.9. Synthesis of 6,11 - OH end-capped Cu(I) bis(NHC) complex

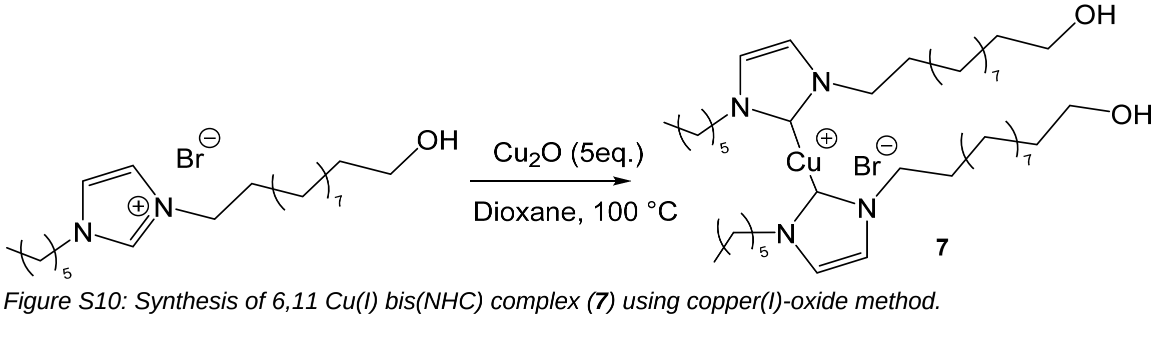

*Figure S10: Synthesis of 6,11 Cu(I) bis(NHC) complex (**7**) using copper(I)-oxide method.*

3-(11-Hydroxyundecyl)-1-hexyllimidazolium bromide (1.20 g, 3.00 mmol) and copper(I)-oxide (2.15 g, 15.00 mmol, 5 eq.) were suspended in dioxane (20 mL) and stirred at 100°C for 3 days. After cooling to RT, the excess of Cu$_2$O was filtered off and the solvent was removed

under reduced pressure. Compound **7** (0.66 g, 0.84 mmol, 28%) was obtained after column chromatography on silica by gradually changing the polarity of solvent from pure $CHCl_3$ to $CHCl_3$/MeOH 20/1 (R_f = 0.40 $CHCl_3$/MeOH 20/1) as a colorless solid.

<u>^{1}H-NMR ($CDCl_3$, 400 MHz):</u> δ [ppm] 6.16 (s, 4H, NC*H*C*H*N), 3.63 (q, $^3J_{H,H}$ = 6.5 Hz, 4H, OC*H₂*), 3.58 (t, $^3J_{H,H}$ = 7.3 Hz, 8H, NCH_2), 1.70 – 1.60 (m, 8H, NCH_2CH_2), 1.56 (p, $^3J_{H,H}$ = 6.8 Hz, 4H, OCH_2CH_2), 1.37 – 1.20 (m, 40H, $(CH_2)_{20}$), 0.87 (t, $^3J_{H,H}$ = 6.5 Hz, 6H, CH_3).

<u>^{13}C-NMR ($CDCl_3$, 100 MHz):</u> δ [ppm] 152.9 (N*C*CuN), 109.9 (CH_3N*C*H*C*HN), 62.9 (O*C*H_2), 43.4 (2xN*C*H_2), 32.8, 31.4, 29.6, 29.5, 29.5, 29.4, 29.3, 29.1, 26.6, 26.3, 25.7, 22.5, 14.0 (*C*H_3).

<u>ESI-TOF MS (positive mode, THF, *m/z*):</u> [M-Br]$^+$ found 707.548, simulated 707.526 for $C_{40}H_{76}CuN_4O_2^+$.

NMR and ESI Spectra

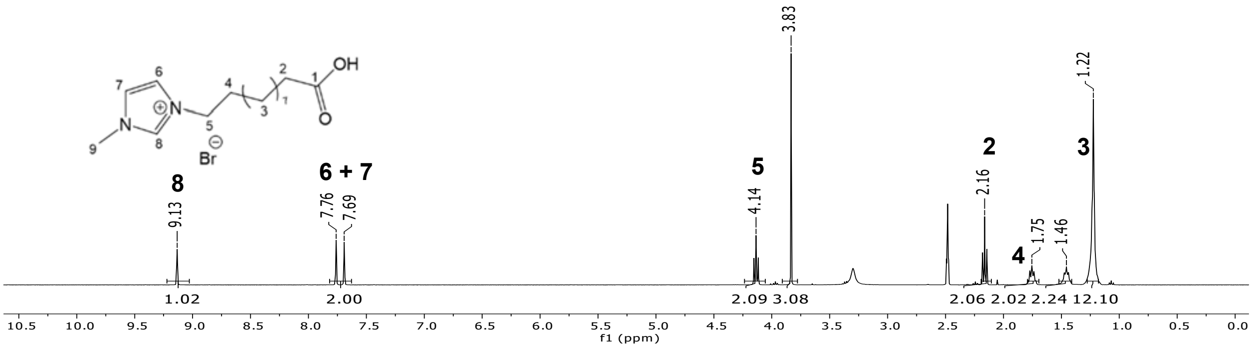

*Figure S11: ^{1}H–NMR spectrum of **5** in DMSO–d_6.*

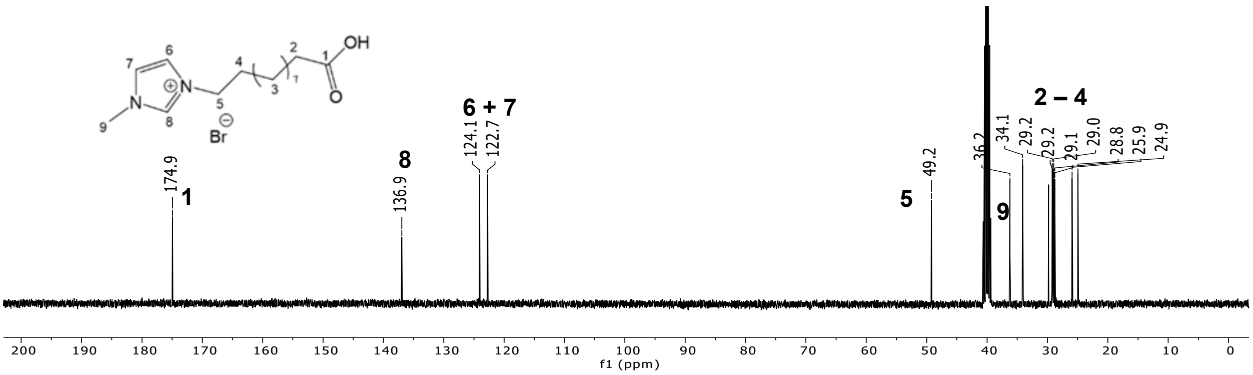

*Figure S12: ^{13}C–NMR spectrum of **5** in DMSO–d_6.*

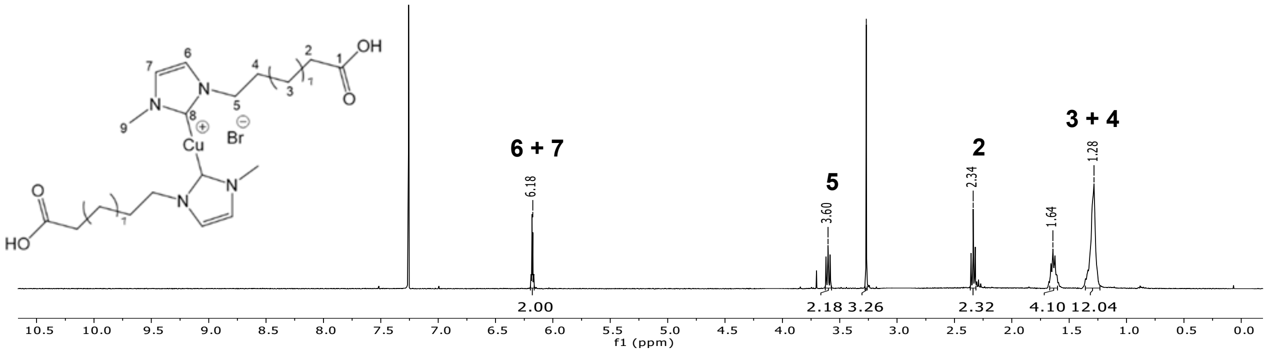

*Figure S13: ^{1}H–NMR spectrum of **6** in $CDCl_3$.*

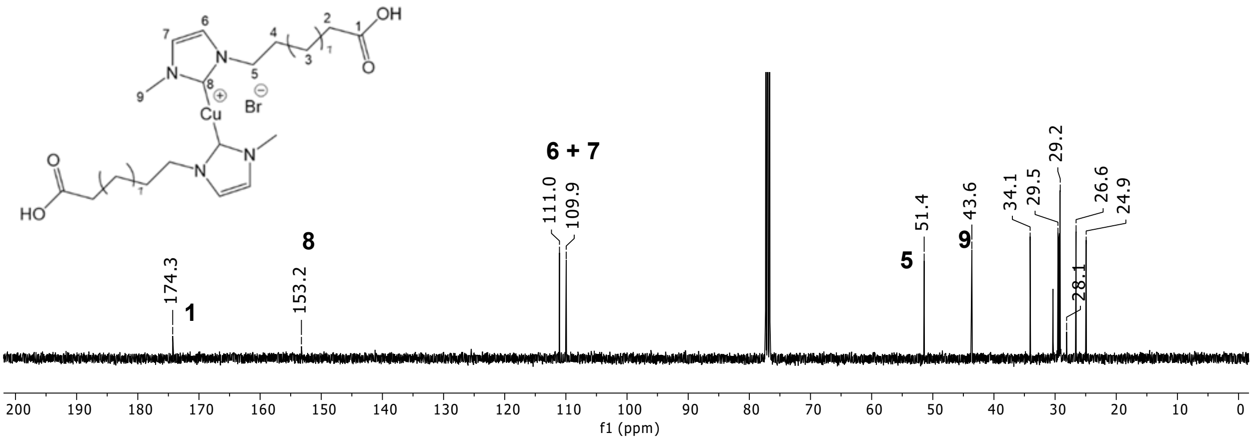

*Figure S14: ^{13}C–NMR spectrum of **6** in $CDCl_3$.*

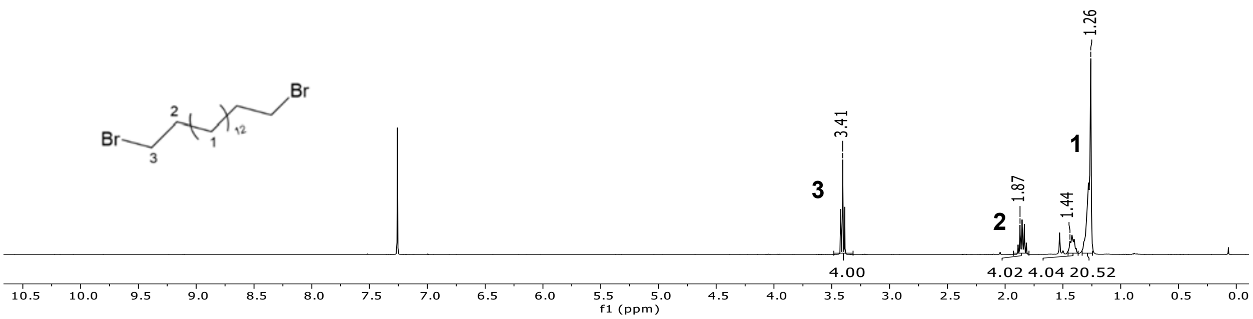

Figure S15: ^{1}H–NMR spectrum in $CDCl_3$.

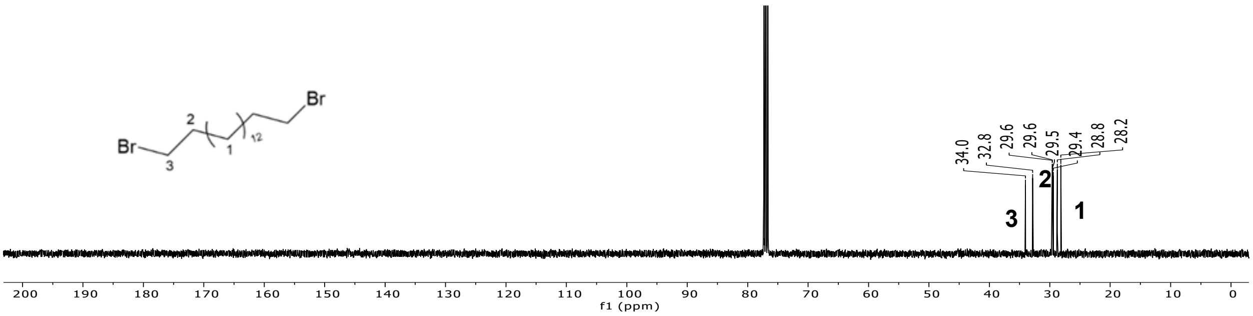

Figure S16: ^{13}C–NMR spectrum in $CDCl_3$.

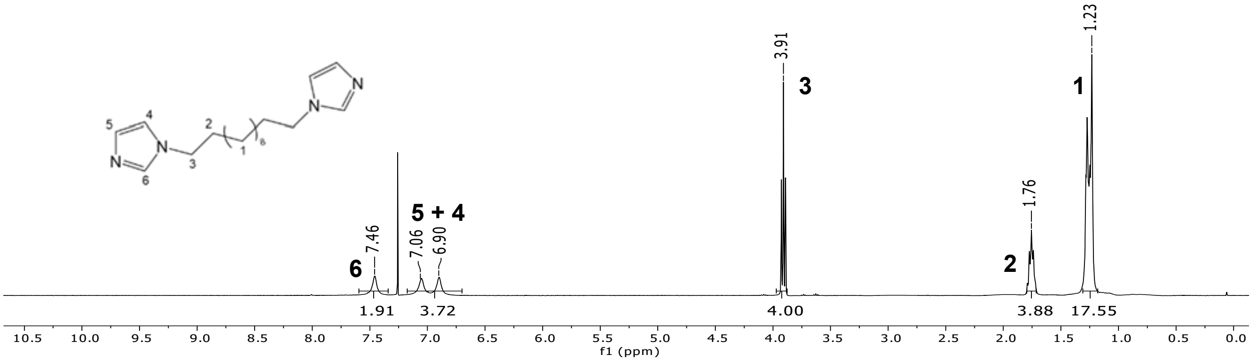

*Figure S17: ^{1}H–NMR spectrum of **4a** in $CDCl_3$.*

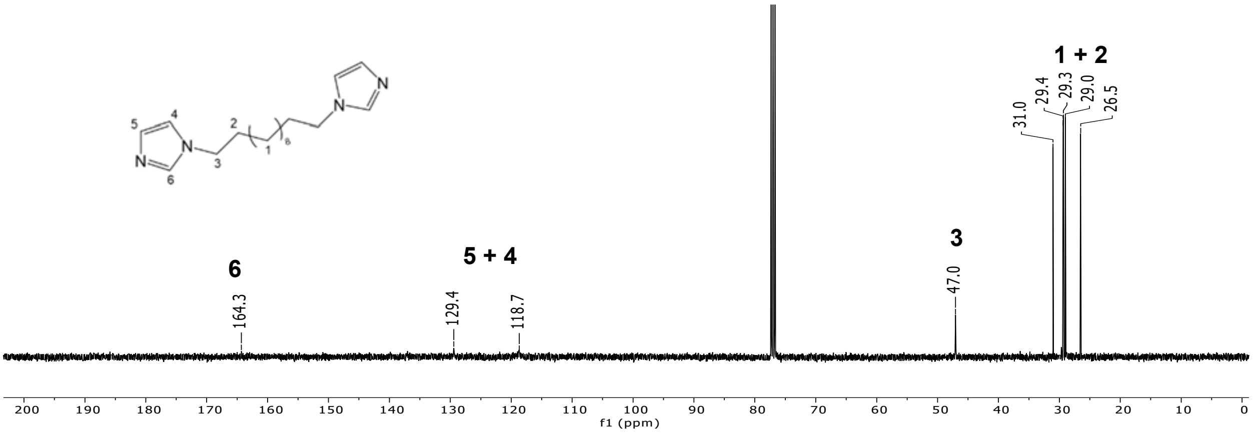

*Figure S18: ^{13}C–NMR spectrum of **4a** in $CDCl_3$.*

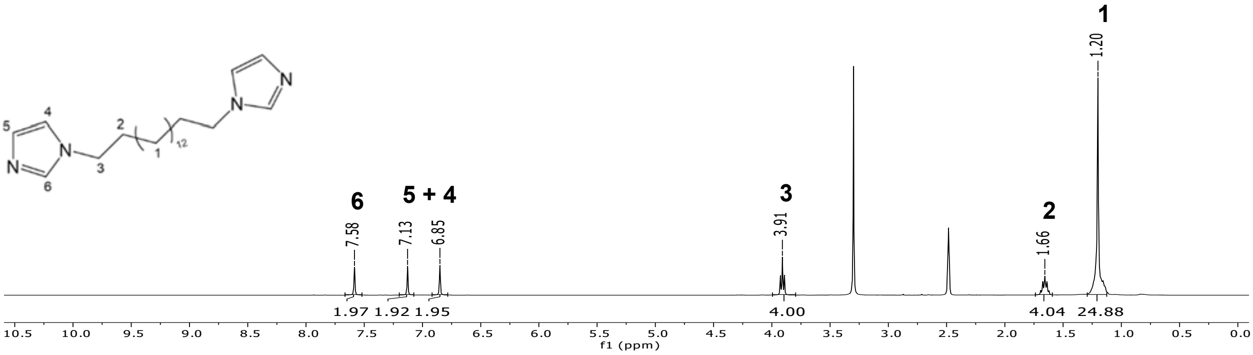

*Figure S19: ^{1}H–NMR spectrum of **4b** in DMSO–d_6.*

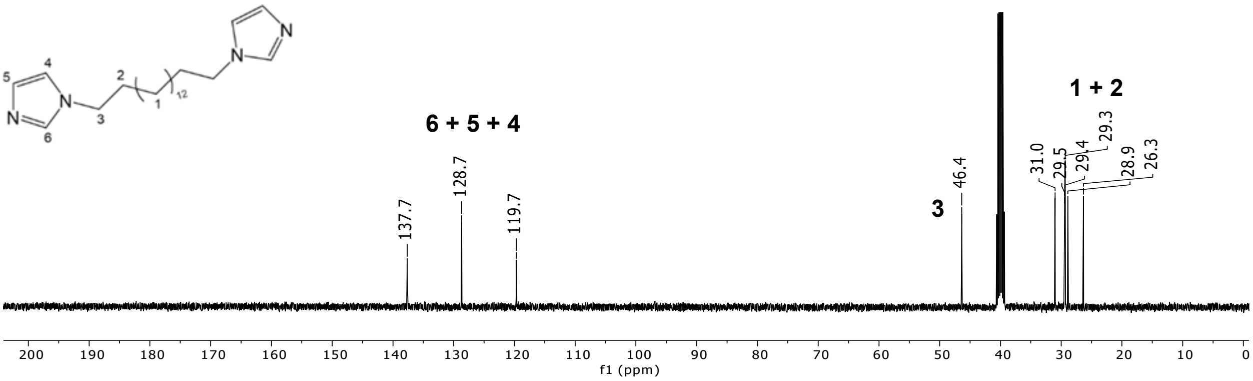

*Figure S20: ^{13}C–NMR spectrum of **4b** in DMSO–d_6.*

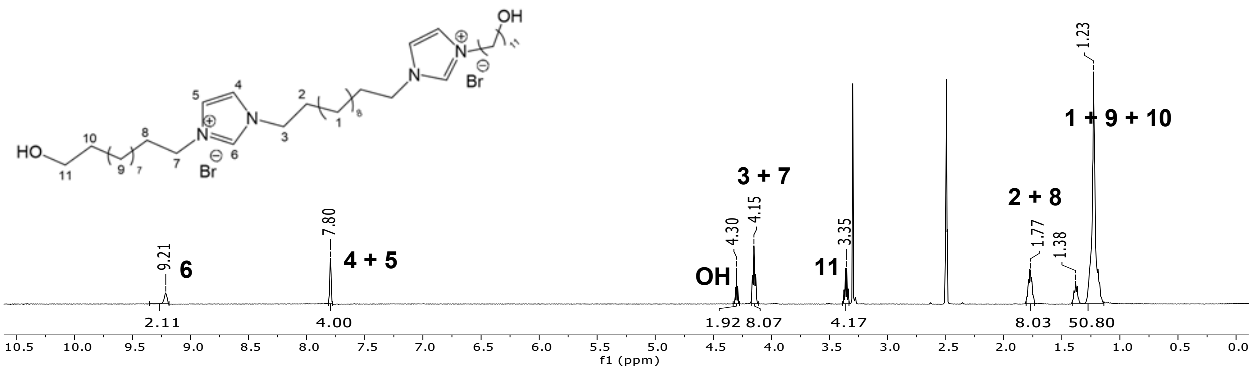

*Figure S21: ^{1}H–NMR spectrum of **3a** in DMSO–d_6.*

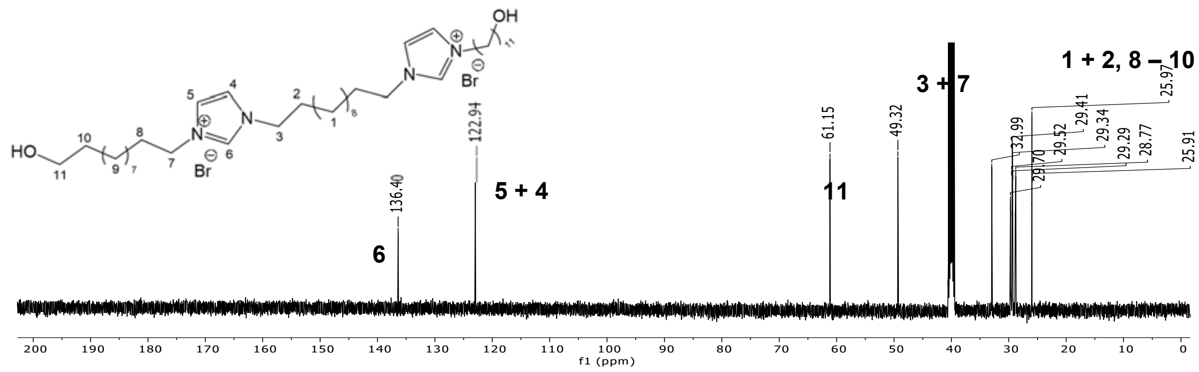

Figure S22: ^{13}C–NMR spectrum of **3a** in DMSO–d_6.

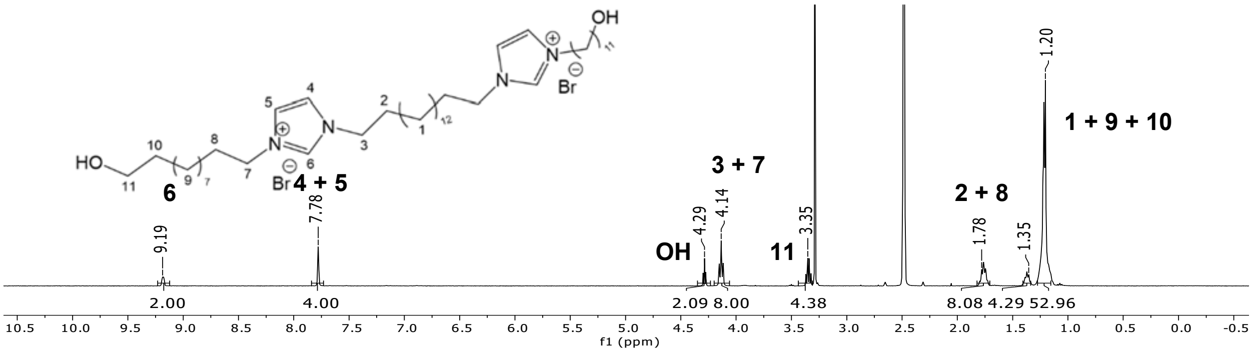

Figure S23: ^{1}H–NMR spectrum of **3b** in DMSO–d_6.

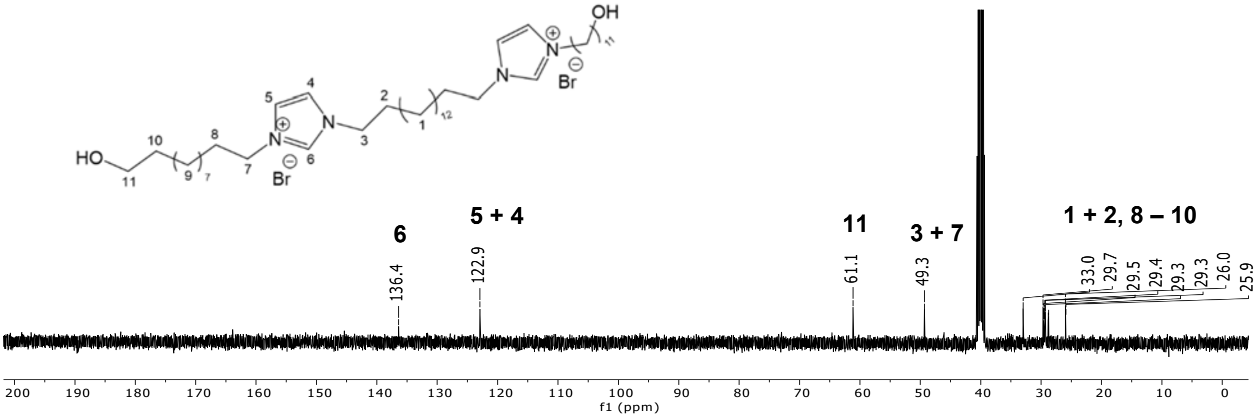

Figure S24: ^{13}C–NMR spectrum of **3b** in DMSO–d_6.

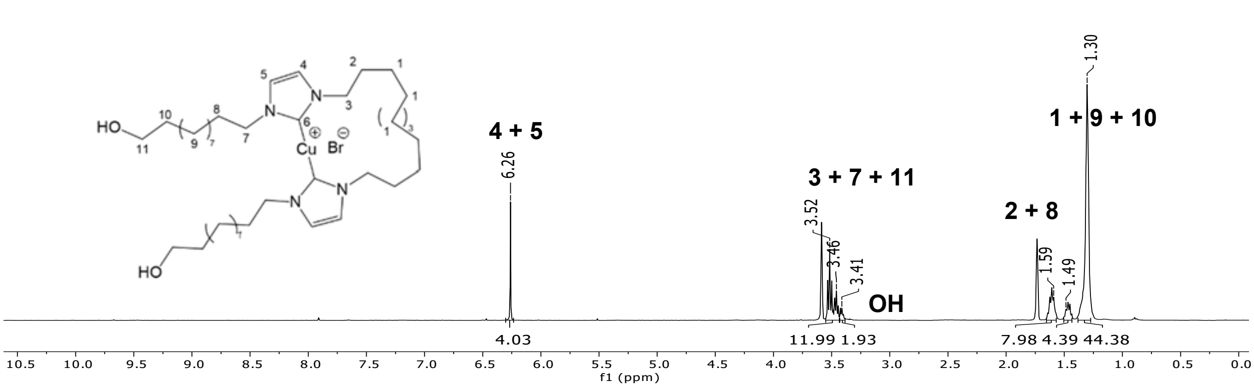

Figure S25: ^{1}H–NMR spectrum of **2a** in THF–d_8.

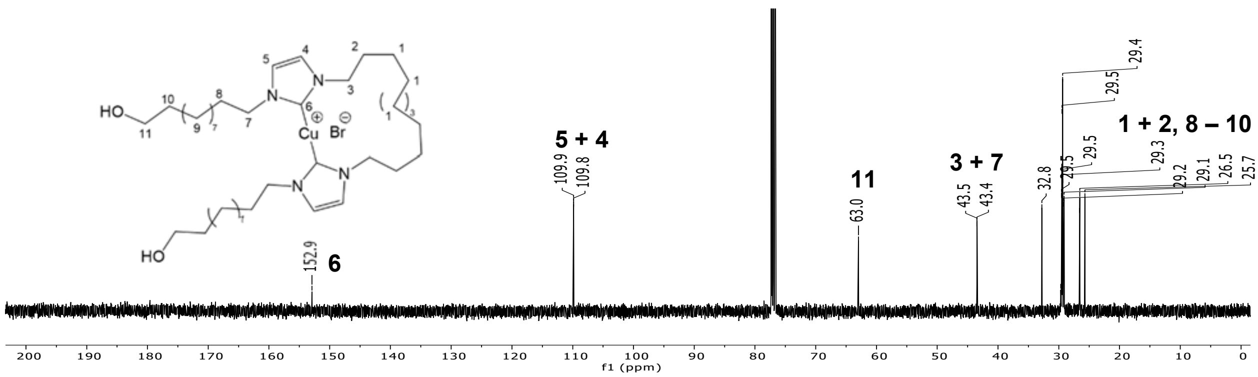

*Figure S26: ^{13}C–NMR spectrum of **2a** in THF–d_8.*

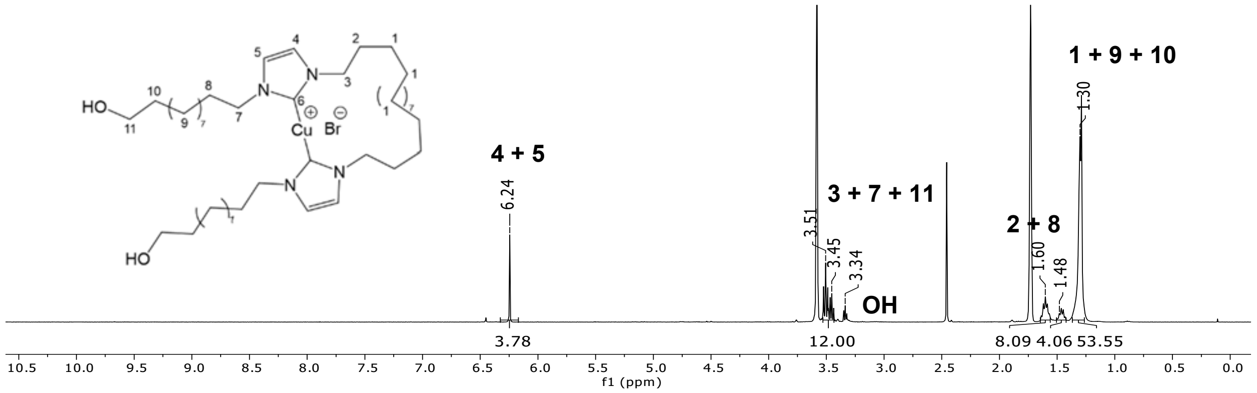

*Figure S27: ^{1}H–NMR spectrum of **2b** in THF–d_8.*

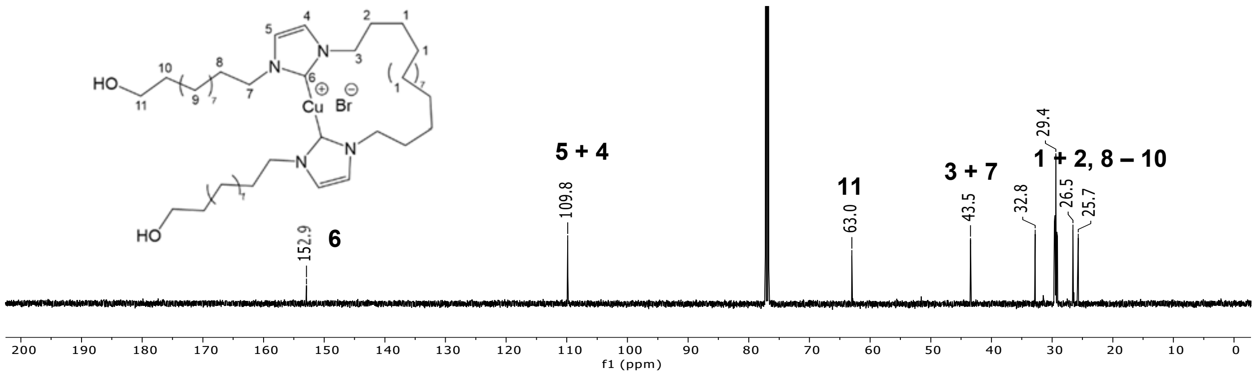

*Figure S28: ^{13}C–NMR spectrum of **2b** in THF–d_8.*

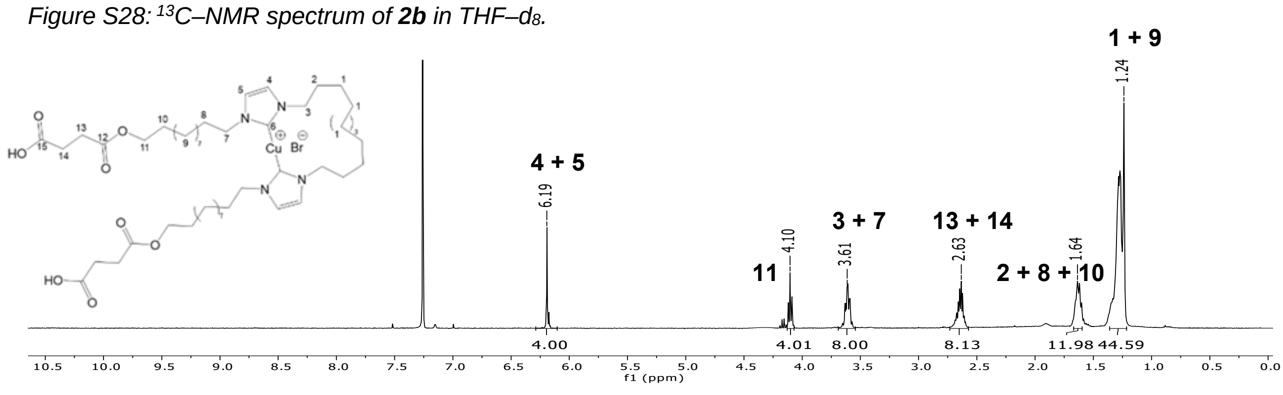

*Figure S29: ^{1}H–NMR spectrum of **1a** in $CDCl_3$.*

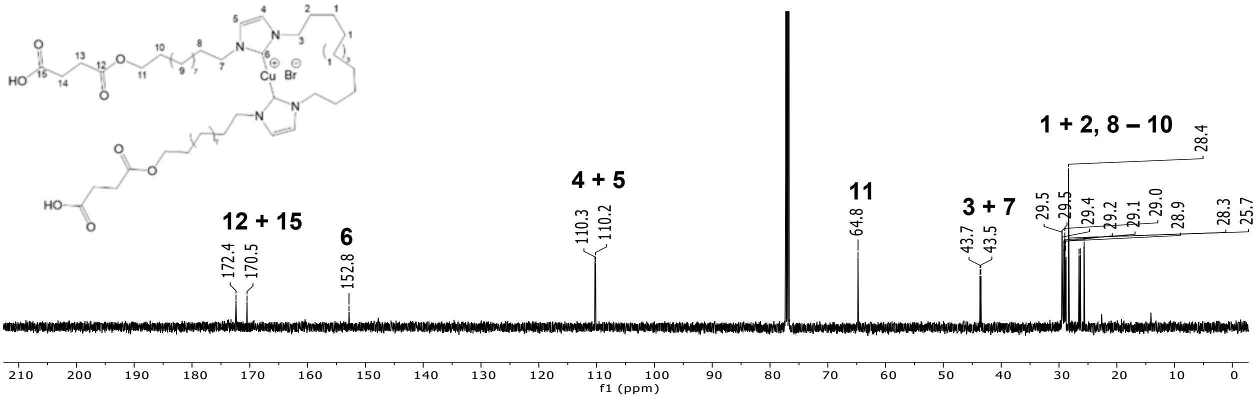

*Figure S30: ^{13}C–NMR spectrum of **1a** in $CDCl_3$.*

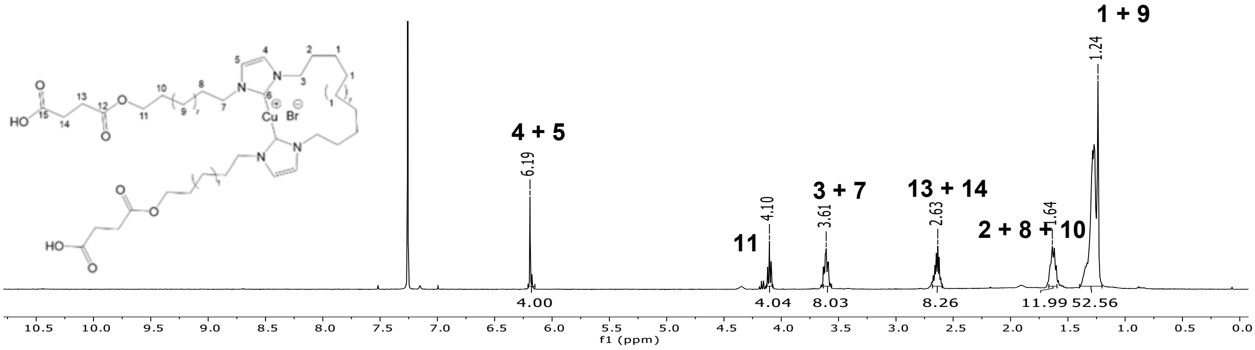

*Figure S31: ^{1}H–NMR spectrum of **1b** in $CDCl_3$.*

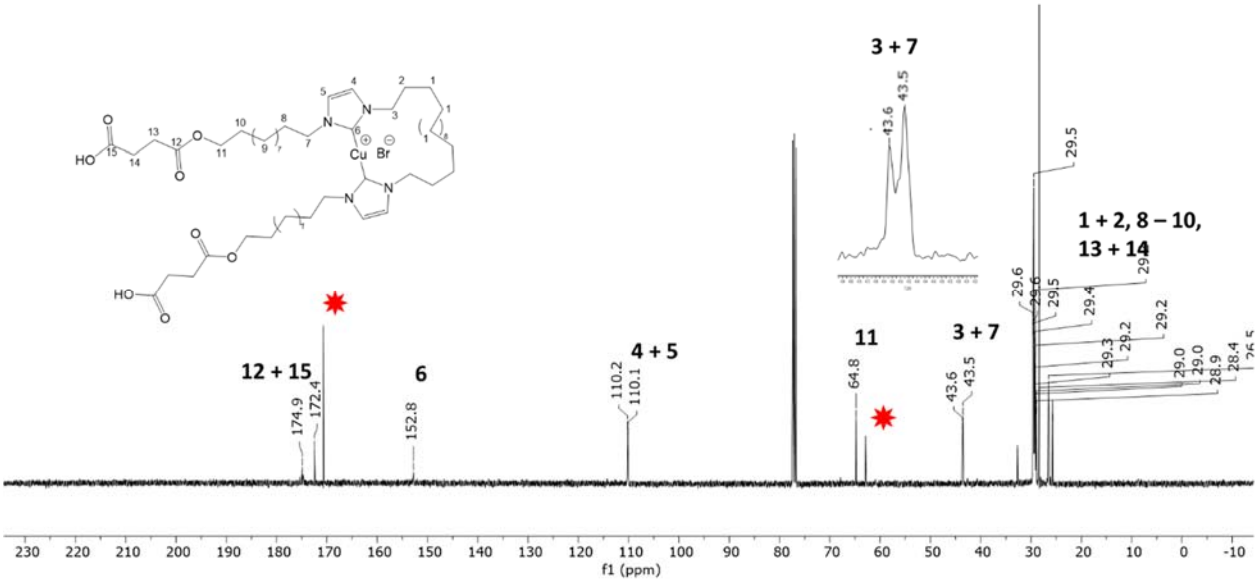

*Figure S32: ^{13}C–NMR spectrum of **1b** in $CDCl_3$. Red stars represent impurities, possibly originating from decomposition.*[1]

[1] Recorded using remaining sample not used for experiments. Due to lack of substance, a purified spectrum could not be recorded.

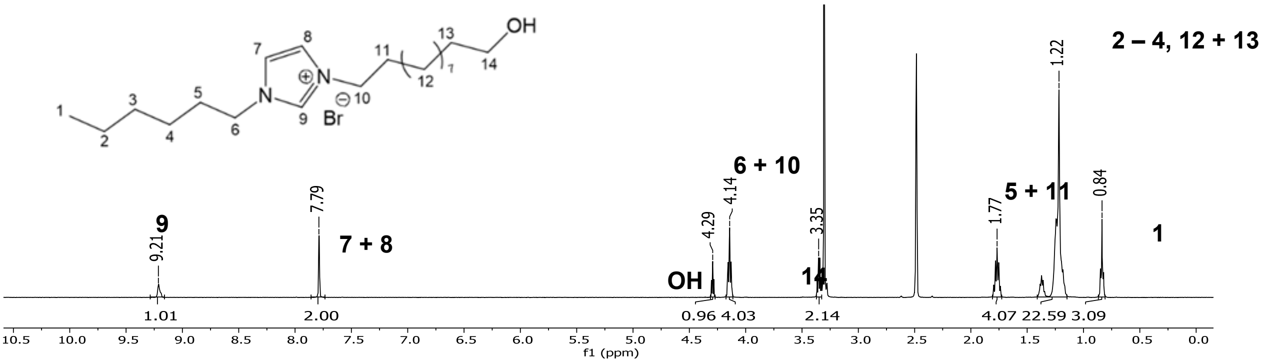

Figure S33: ^{1}H–NMR spectrum in DMSO–d6.

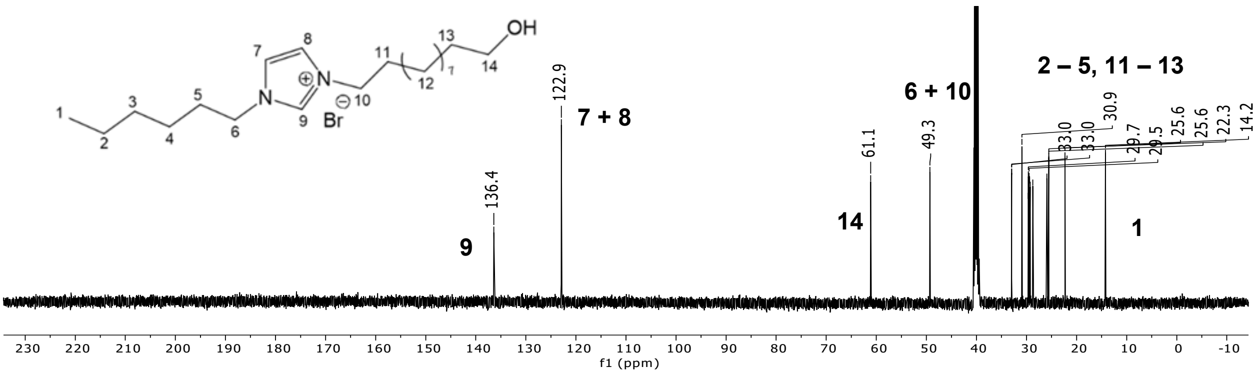

Figure S34: ^{13}C–NMR spectrum in DMSO–d6.

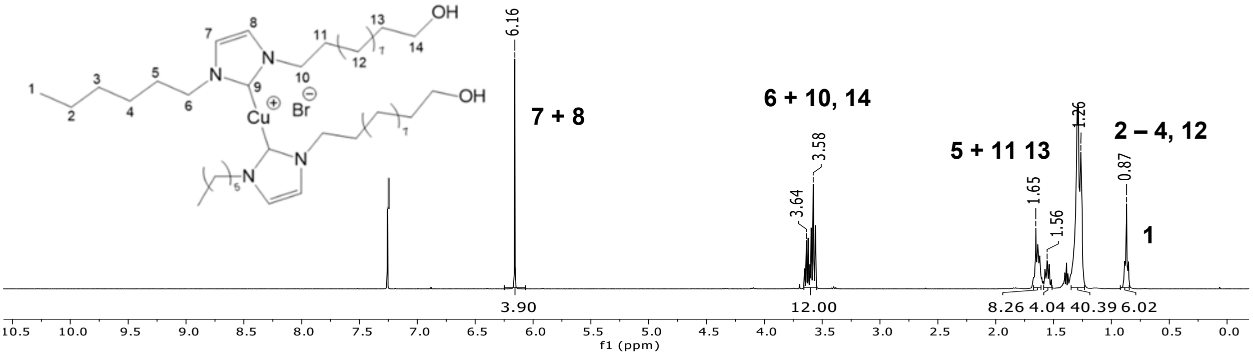

*Figure S35: ^{1}H–NMR spectrum of **7** in CDCl3.*

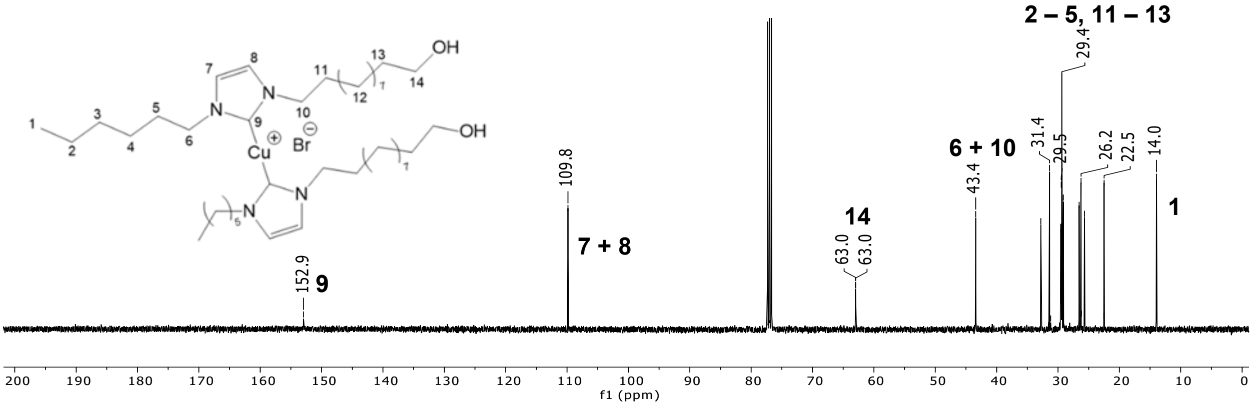

*Figure S36: ^{13}C–NMR spectrum of **7** in CDCl3.*

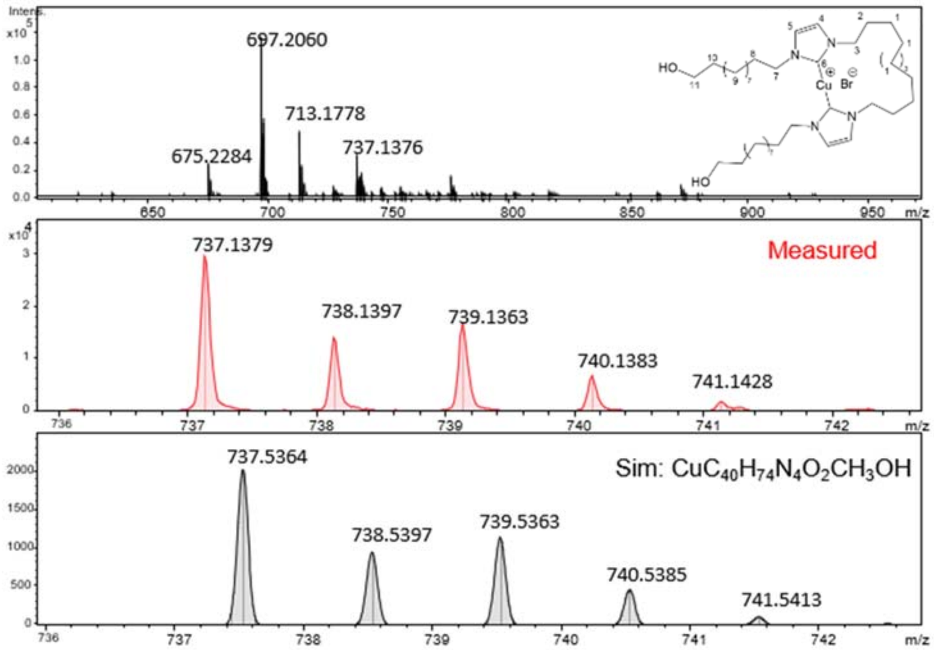

Figure S37: ESI–TOF–MS spectrum of ***2a.*** *In this experiment, mass calibration was significantly off, the measured masses are not possible for singly charged ions composed of the elements present in the sample. The assignment is based on the characteristic isotope pattern, for which perfect agreement is reached.*

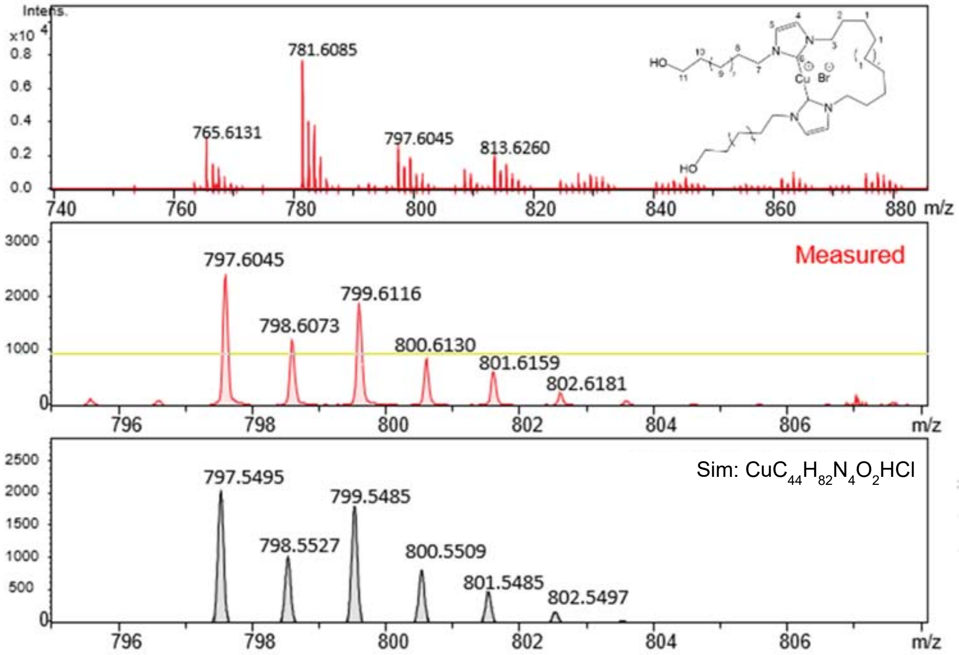

*Figure S38: ESI–TOF–MS spectrum of **2b.***

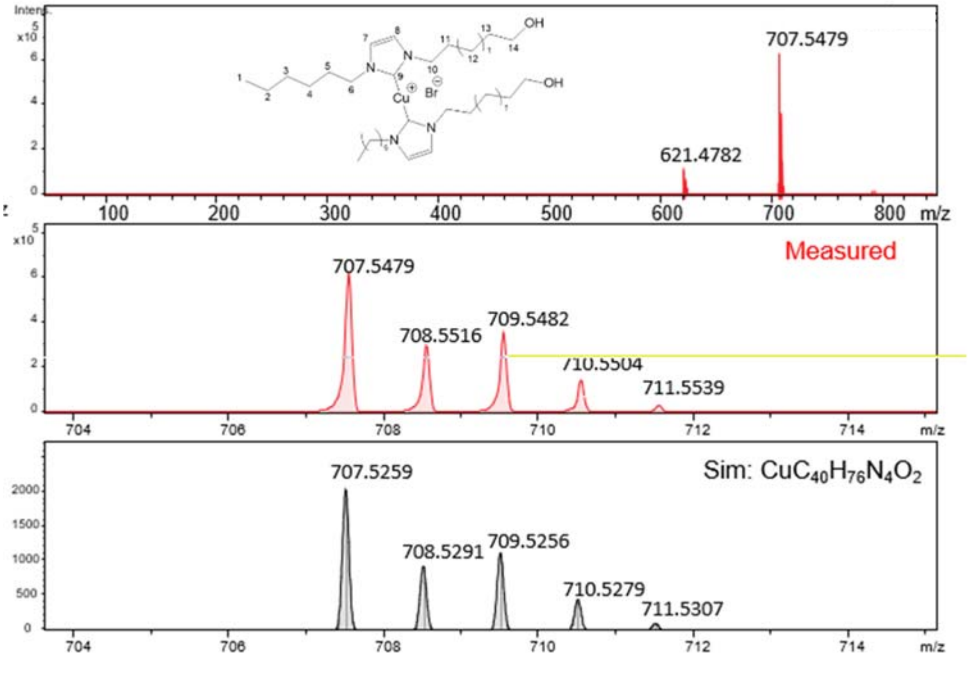

*Figure S39: ESI–TOF–MS spectrum of **7.***

II) Single-Molecule Force Spectroscopy

Consumables / Chemicals

As substrate surface for SMFS experiments conventional microscope slides (Carl Roth) were applied. Commercial silicon nitride cantilevers (MLCT, cantilever E; Bruker AFM Probes) with a nominal spring constant of 100 mN m^{-1} were chosen for their high sensitivity and acceptable noise level, necessary for determining characteristic rupture events. Silane polyethylene glycol amine (SPA) with a molecular weight of 5 kDa (PG2-AMSL-5000) for surface and cantilever functionalization was obtained from Nanocs. Coupling agents *N*-(3-dimethylaminopropyl)-*N*'-ethylcarbodiimide hydrochloride (EDC) and *N*-hydroxysuccinimide (NHS), as well as hydrochloric acid (37%), acetic acid (> 99.8%) and adipic acid were obtained from Merck. Double distilled water, ethanol (> 99.8%) and dimethyl sulfoxide (DMSO; > 99.8%) was acquired from Carl Roth.

Functionalization

Prior to functionalization, cantilevers and glass surfaces were cleaned separately. Cantilevers were irradiated with UV light using a UV PenRay (Ultra-Violet Products Ltd, Cambridge, UK, Model 11SC-1) for two hours. Glass surfaces in a dye chamber filled with diluted hydrochloric acid (3.7%) were placed in an ultrasonic bath for 90 minutes. Subsequently, the surfaces were washed and sonicated three more times for 10 minutes in water (double distilled). Surfaces and cantilevers were then washed in ethanol.

For the functionalization solution, 1.0–1.5 mg SPA were distributed into 10 eppendorf tubes to minimize agglomeration and 1 mL water was added to each tube. The tubes were then mixed for 10 minutes at 3000 rpm using a vortex shaker. Furthermore, 90 mL ethanol were acidized using diluted acetic acid (10%) to pH 5 (± 0.5). The SPA solutions were then added to the ethanol and thoroughly mixed.

Cantilevers and glass surfaces were then placed in the solution for 90 minutes, washed twice in water and twice in ethanol. They were then dried in the oven for one hour at 60°C, left to cool in the oven and stored in a desiccator with silica gel under vacuum (maximum two weeks, even though no degradation was observed).

Experiment

To allow SMFS experiments with copper complexes or adipic acid (in the following denoted as "sample"), the SPA-functionalized glass surface had to be activated. To this aim, two solutions were prepared, one containing the sample and one the coupling agents NHS and EDC. As solvent the reaction medium DMSO was used. Typically, 1.0–1.5 mg of the sample was solvated in 0.2 mL DMSO for solution 1, 4–5 mg NHS and 10–12 mg EDC in 0.7 mL DMSO

for solution 2. Both solutions were mixed in a vortex shaker for 10 min @ 3000 rpm. Subsequently, 0.4 mL of solution 2 was added to solution 1 to activate the carboxylic groups. The solutions were mixed for another 10 min.

Meanwhile, the glass surface was covered with water for 20 min, rinsed and acidized using diluted acetic acid (2M) for 3 min. Subsequently, it was rinsed twice with water and twice with DMSO. After each following step, the surface was rinsed twice with DMSO and dried on the edges with a paper towel (Kimtech). Next, the surface was covered with solution 1, containing the activated sample and kept for 10 min. This step was repeated a second time. To ensure activation of the surface species, the glass slide was covered with solution 2 containing only the coupling agents for 10 more minutes.

Prior to measurement, the functionalized cantilever was calibrated in DMSO. An indentation curve was recorded on a cleaned, non-functionalized glass surface and the sensitivity of the cantilever determined. The spring constant was obtained by measuring the thermal noise 200 µm from the surface over 30 s. This procedure was repeated three times as well. The average of the obtained values was used.

For measurements, force distance curves were recorded on 40 x 40 points on a 100 x 100 µm map. Gains were adjusted to the feedback loop and cantilever approached to a setpoint of 0.25 nN. The cantilever dwelled for 3 s at the surface and was retracted with a velocity 1.0 µm s^{-1} over a length of 1 µm. Sample rate during retract was chosen at 40 kHz to allow sufficient data points for identification of characteristic double rupture events. All experiments were carried out using a NanoWizard 4 AFM (JPK Instruments, Bruker).

Data Analysis

Data evaluation was carried out without knowledge of which sample was used during the measurements as to not be biased during the evaluation. The samples used were adipic acid, copper biscarbene complexes without safety line and those with a safety line consisting of 12 or 16 CH_2 groups.

Force distance curves were pre-selected using a filter in the JPK Data Processing Software to determine those showing possible rupture events of covalent bonds (forces higher 500 pN). Subsequently, curves showing characteristics of double rupture events, viz. almost identical slopes before and after the event and elongations between 0.5 and 50 nm, were selected. Data from these curves was then fitted to trendlines to accurately compare the slopes and calculate the elongation at the determined rupture force. Error estimation of the elongation was performed using gaussian error propagation including correlation, while a commonly acknowledged error of 20% for these experiments was assumed for rupture forces.

Table S1: Identified double rupture events and corresponding samples. Given are rupture forces [pN] measured for the characteristic event, calculated elongation [nm], slopes [pN/nm] from obtained fits before (s1) and after (s2) the rupture event as well as absolute (abs.) [pN/nm] and relative (rel.) [%] errors for the elongation determined using gaussian error propagation

**Event No.** [#]	**safety line** [xCH_2]	**rupture force** F [pN]	**elongation** d [nm]	**slope** s1 [pN/nm]	s2 [pN/nm]	**error (elongation)** abs. [nm]	rel. [%]
1	–	2658	2.14	*117.9*	*116.3*	0.07	3.17%
2	12	1595	1.76	*118.3*	*120.7*	0.07	3.73%
3	12	2114	1.87	*157.5*	*153.8*	0.07	3.57%
4	12	2607	1.33	*166.5*	*164.6*	0.04	2.81%
5	12	2100	1.6	*114.5*	*113.0*	0.06	3.85%
6	12	1579	1.41	*119.6*	*122.7*	0.07	4.97%
7	12	1611	1.57	*127.0*	*126.3*	0.03	1.72%
8	12	1723	1.22	*122.6*	*124.2*	0.05	4.07%
9	12	1691	2.39	*161.8*	*166.2*	0.06	2.38%
11	12	2293	1.41	*141.8*	*142.8*	0.04	2.69%
12	12	1584	2.09	*137.4*	*140.6*	0.06	2.89%
13	16	2342	3.39	*135.1*	*137.6*	0.07	1.92%
14	16	1874	2.79	*141.0*	*142.3*	0.03	1.20%
15	16	2066	1.92	*174.7*	*168.5*	0.09	4.55%
16	16	1999	1.53	*152.2*	*154.7*	0.05	3.16%
17	16	2164	2.85	*137.0*	*133.8*	0.08	2.77%
18	16	2416	3.50	*150.0*	*149.1*	0.03	0.76%
19	16	2062	1.40	*140.1*	*140.1*	0.03	2.21%
20	16	1997	1.54	*154.3*	*152.1*	0.04	2.87%
21	16	1738	2.08	*159.0*	*161.5*	0.04	1.80%
22	16	2398	1.77	*156.9*	*157.6*	0.03	1.42%
23	16	2011	2.90	*142.3*	*142.3*	0.02	0.55%

Illustration of a threaded structure

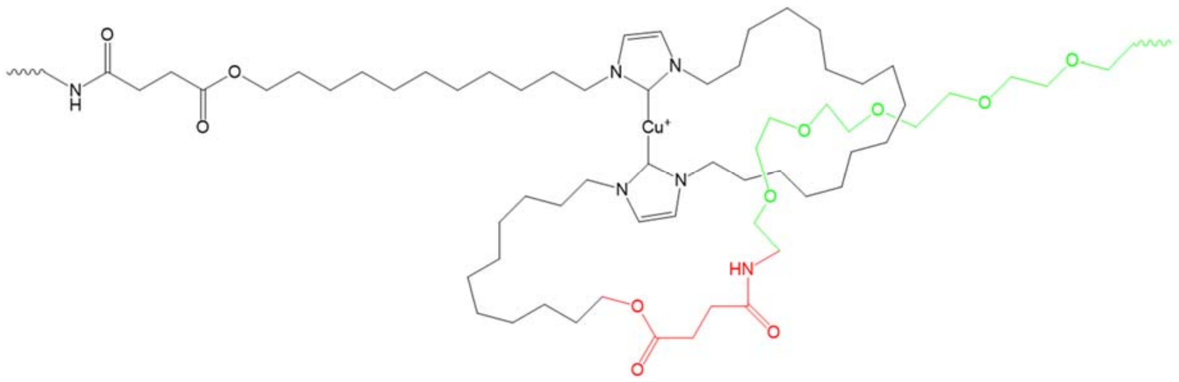

Figure S40: Suggested formation of a threaded structure. The amine-terminated PEG (green) slips through the macrocycle before the amide bond forms. We speculate that the amide and ester units (red) are too bulky and do not fit through the macrocycle, which prevents the disentanglement upon stretching.

III) Computation

To get a general estimation of rupture forces and elongations expected from the measurements, quantum chemical calculations were carried out. Using the COGEF method (Constrained Geometry for simulating external Force), the application of external force on the mechanophore is simulated, yielding maximum rupture forces and elongation upon rupture. Smaller model molecules with shorter side chain and safety line were used to limit necessary computation power while allowing DFT calculations at B3LYP/6-31+g* level of theory. By increasing the distance between terminal C atoms in small steps and optimizing the geometry after each step, energy and necessary force can be calculated dependent on the distance of the fixed C atoms, given in Figure S40.

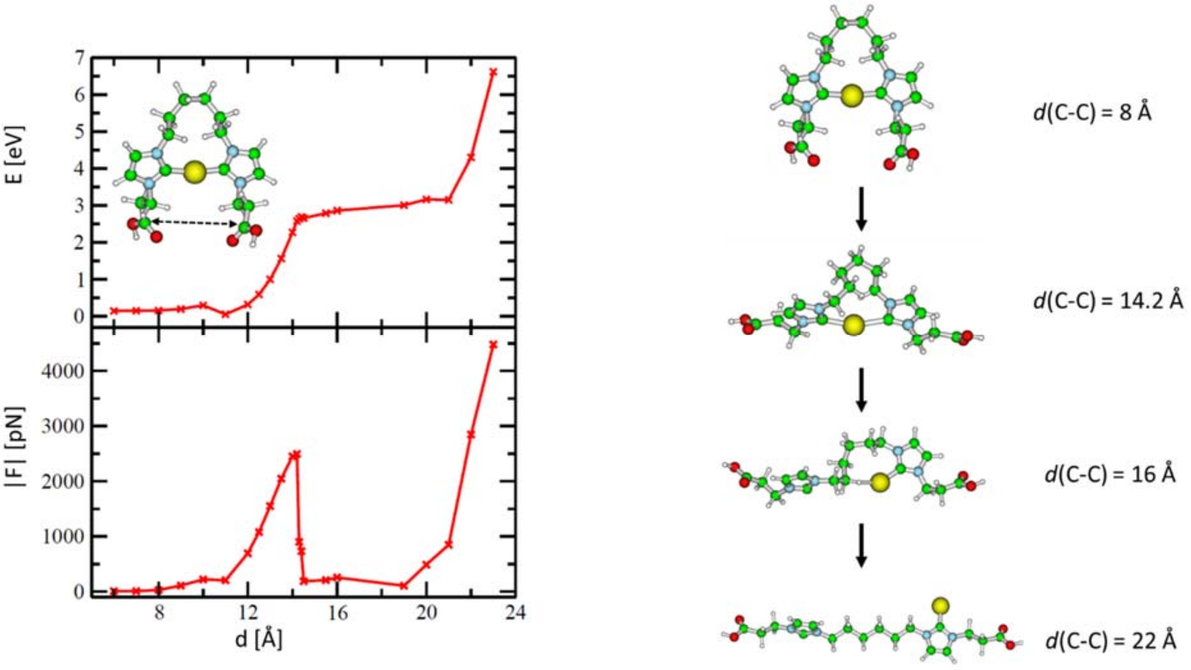

Figure S41: Left: Energy E in eV (top) and absolute force |F| in pN (bottom) dependent on the distance d of the terminal C atoms of the model molecule after optimization, calculated at the B3LYP/6-31+g level of theory. Right: Structure of model molecule (yellow: Cu; green: C; turquoise: N; red: O; white: H) dependent on the distance of the terminal C atoms, given for 8, 14.2, 16 and 22 Å.*

Calculations using B3LYP/6-31g* and PM6 level of theory yielded similar results, as shown for a model molecule with a safety line consisting of 6 CH_2 groups, given in Figure S41 (top diagrams). Furthermore, calculated curves for longer safety lines (PM6) are given, showing the elongations assumed for our experiment to be around 1.6 nm for the short and 2.1 nm for the longer safety line, respectively.

B3LYP/6-31G*

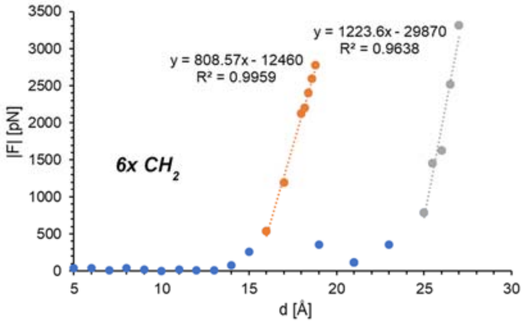

Figure S42: Calculated force-distance curves using B3LYP/6-31g (left) and PM6 (right) level of theory. For B3LYP/6-31g*, COGEF calculations were carried out using a safety line consisting of 6 CH_2 groups. For PM6, COGEF simulations of model molecules with 6 (top), 12 (middle) and 16 CH_2 groups (bottom) were used. Maximum rupture forces were calculated to be around 2500–3000 pN, elongations about 0.8, 1.6 and 2.1 nm for the different safety line lengths, respectively.*

PM6

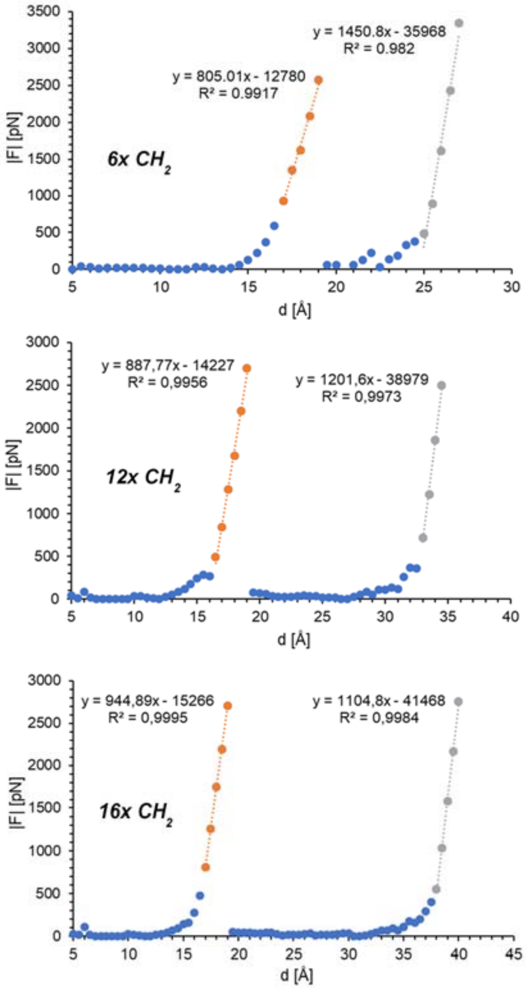

Supplement: Supplementary file 1 — Supplementary [file CHEM-27-8723-s001.pdf]
